# Tuning the size and composition of manganese oxide nanoparticles through varying temperature ramp and aging time

**DOI:** 10.1371/journal.pone.0239034

**Published:** 2020-09-18

**Authors:** Celia Martinez de la Torre, Jasmine H. Grossman, Andrey A. Bobko, Margaret F. Bennewitz

**Affiliations:** 1 Department of Chemical and Biomedical Engineering, West Virginia University, Morgantown, WV, United States of America; 2 Department of Biochemistry and In Vivo Multifunctional Magnetic Resonance Center, West Virginia University, Morgantown, WV, United States of America; Chung-Ang University College of Engineering, REPUBLIC OF KOREA

## Abstract

Manganese oxide (MnO) nanoparticles (NPs) can serve as robust pH-sensitive contrast agents for magnetic resonance imaging (MRI) due to Mn^2+^ release at low pH, which generates a ~30 fold change in T_1_ relaxivity. Strategies to control NP size, composition, and Mn^2+^ dissolution rates are essential to improve diagnostic performance of pH-responsive MnO NPs. We are the first to demonstrate that MnO NP size and composition can be tuned by the temperature ramping rate and aging time used during thermal decomposition of manganese(II) acetylacetonate. Two different temperature ramping rates (10°C/min and 20°C/min) were applied to reach 300°C and NPs were aged at that temperature for 5, 15, or 30 min. A faster ramping rate and shorter aging time produced the smallest NPs of ~23 nm. Shorter aging times created a mixture of MnO and Mn_3_O_4_ NPs, whereas longer aging times formed MnO. Our results indicate that a 20°C/min ramp rate with an aging time of 30 min was the ideal temperature condition to form the smallest pure MnO NPs of ~32 nm. However, Mn^2+^ dissolution rates at low pH were unaffected by synthesis conditions. Although Mn^2+^ production was high at pH 5 mimicking endosomes inside cells, minimal Mn^2+^ was released at pH 6.5 and 7.4, which mimic the tumor extracellular space and blood, respectively. To further elucidate the effects of NP composition and size on Mn^2+^ release and MRI contrast, the ideal MnO NP formulation (~32 nm) was compared with smaller MnO and Mn_3_O_4_ NPs. Small MnO NPs produced the highest amount of Mn^2+^ at acidic pH with maximum T_1_ MRI signal; Mn_3_O_4_ NPs generated the lowest MRI signal. MnO NPs encapsulated within poly(lactide-co-glycolide) (PLGA) retained significantly higher Mn^2+^ release and MRI signal compared to PLGA Mn_3_O_4_ NPs. Therefore, MnO instead of Mn_3_O_4_ should be targeted intracellularly to maximize MRI contrast.

## Introduction

The use of metal oxide nanoparticles (NPs) has been increasing over the past decades due to their magnetic, electric, and catalytic properties. Of particular interest to biomedical applications is the ability of metal oxide NPs, such as iron oxide and manganese oxide (MnO), to serve as contrast agents for magnetic resonance imaging (MRI) [1]. Typically, the metal oxide crystals are encapsulated within a polymer to promote hydrophilicity and biocompatibility. Iron oxide NPs are superparamagnetic and cause dark contrast on T_2_ and T_2_* MRI. The negative contrast of iron oxide NPs is so robust that even single cells can be visualized on MRI if each cell accumulates at least 1 pg of iron [2–4]. However, iron oxide NPs elicit strong MRI signal in their intact form and therefore constantly generate contrast, or are always in the “ON” state. Furthermore, naturally occurring iron present inside the liver, spleen, bone marrow and blood leads to dark contrast that can be difficult to differentiate from applied iron oxide NPs. As an alternative, manganese oxide (MnO) NPs provide the advantage over iron oxide in that they can provide switchable, bright contrast on T_1_ MRI due to the paramagnetic properties of the Mn^2+^ ion. Our group and other studies have shown that intact MnO NPs are in an “OFF” state and create minimal T_1_ MRI signal due to the Mn^2+^ ions being tightly bound and inaccessible to the surrounding water molecules [5–10]. In acidic media, MnO dissolves to form Mn^2+^, which coordinates with water molecules to decrease T_1_ and produce a positive MRI signal, thus turning “ON” MRI contrast [5–10].

Compared to gold standard and pH-sensitive gadolinium T_1_ MRI contrast agents, MnO NPs have superior MRI properties. Clinically used gadolinium chelates are not pH-sensitive, and are always in an “ON” state, which highlights any well vascularized structure and can lead to false positive diagnoses in which a benign tumor can be mistaken for a malignant tumor [11–13]. In addition, many standard gadolinium agents such as MultiHance have low relaxivities of ~4 mM^-1^s^-1^ at 1.5T-4.7T [14]; Mn^2+^ has a higher relaxivity of ~ 7–8 mM^-1^s^-1^ at the same field strengths [5, 15, 16]. Furthermore, when gadolinium agents are altered to be pH sensitive, T_1_ relaxivity changes only ~2–4 times [17, 18] over pH 5 to 7.4. Polymeric MnO NPs are more powerful smart contrast agents, producing a relaxivity change of ~30 times, as intact NPs have very low r_1_ (0.12–0.21 mM^-1^s^-1^) [5, 19] at pH 7.4 and release Mn^2+^ at pH 5 to increase relaxivity to 7 mM^-1^s^-1^. MnO NPs with targeting agents can be utilized for enhanced specificity for detection of cancerous tumors through NP dissolution inside tumor cells within low pH endosomes or lysosomes.

To enhance MRI signal generation, it is necessary to fine-tune synthesis strategies to control and reduce the size of MnO NPs. It was hypothesized that smaller NP diameters will increase the surface area to volume ratio to facilitate faster dissolution of MnO to Mn^2+^ to generate higher MRI signal under acidic conditions and allow for more efficient packing of MnO NPs into polymeric or liposomal delivery systems. MnO NPs are commonly synthesized by thermal decomposition of a manganese-based compound such as manganese acetylacetonate (Mn(II) ACAC) [20–22], Mn oleate [23], Mn acetate [21, 24–26], Mn carbonate [27] or Mn stearate [28]. Several different variables can be modified to optimize the physical and chemical properties of the synthesized MnO NPs including the type of inert gas [20–22], peak reaction temperature [21–23, 26], total reaction time [23, 24, 28], and types/ratios of initial chemical compounds [20–22, 24, 25] utilized in the reaction. To date, the effects of temperature ramp rate and aging time on both size and composition have not been explored. Herein, we systematically evaluate how two temperature ramping rates (10°C/min and 20°C/min) combined with increasing aging times (5, 15 and 30 min) at 300°C can be utilized to synthesize smaller NPs of pure MnO composition. MRI was utilized to evaluate T_1_ signal enhancement of Mn^2+^ ions released from NPs with different sizes and compositions to determine which formulation maximized MRI contrast at pH 5, 6.5 and 7.4.

## Materials and methods

### Chemicals

Mn(II) ACAC, oleylamine (70%, technical grade), poly(vinyl alcohol) (PVA), and rhodamine 6G were obtained from Sigma-Aldrich. Dibenzyl ether (≥99%, Acros Organics), hexane (≥98.5%, Macron Fine Chemicals), dichloromethane (≥99.5% stabilized ACS, BDH Chemicals), Dulbecco's phosphate buffered saline (PBS), sodium citrate dihydrate (BDH Chemicals), citric acid (VWR Chemicals, LLC), and manganese(II) chloride tetrahydrate (98–101% ACS, VWR Chemicals, LLC) were purchased from VWR. Hydrochloric acid (HCl) TraceMetal™ Grade was acquired from Fisher Scientific. Ethanol (Decon Laboratories Inc.) was obtained internally from West Virginia University’s Environmental Health and Safety Services. Ester-terminated 50:50 poly(D,L-lactide-co-glycolide) (PLGA) (inherent viscosity: 0.55–0.75 dL/g) was obtained from Lactel Absorbable Polymers.

### Synthesis of MnO NPs

All work for MnO NP synthesis should be performed under a chemical fume hood with proper PPE including safety glasses, nitrile gloves and a lab coat. MnO NPs were fabricated using a standard thermal decomposition reaction of Mn(II) ACAC dissolved in oleylamine and dibenzyl ether based on the synthesis of magnetite (Fe_3_O_4_) NPs by Xu et al. [29] Mn(II) ACAC (6 mmol) was dissolved in 40 mL of oleylamine and 20 mL of dibenzyl ether. The solution was heated with a heating mantle connected to a thermocouple probe immersed into the reaction mixture and a programmable temperature controller. According to the user defined temperature profile, the mixture was heated from room temperature to 60°C over 30 min under a constant flow of inert N_2_ gas. A constant N_2_ flow was needed to successfully remove all oxygen from the reaction and obtain the desired product, MnO NPs. Then, the temperature was quickly raised to 300°C under N_2_ gas using two different ramp rates of 20°C/min or 10°C/min and aged at 300°C for either 5, 15, or 30 min. To assess variability between synthesized NP batches, each of the 6 different temperature profiles were independently run 3 times, obtaining a total of 18 batches of MnO NPs. All of the 18 batches were utilized for further experiments. Upon completion of the reaction, the heating mantle was removed to allow the solution to cool down to room temperature. The MnO NPs were pelleted in Nalgene® Oak Ridge centrifuge tubes following centrifugation at 17,400 x g for 10 min and washed 4 to 5 times in hexane and ethanol using the same centrifugation procedure. Resulting MnO NPs were resuspended in hexane and left in a fume hood to dry overnight. After overnight drying, the MnO NPs were baked over 24 hr in an oven at 100°C. The resulting MnO NPs synthesized by thermal decomposition were hydrophobic and capped with oleylamine.

### PLGA encapsulation of MnO NPs

For a separate set of experiments, MnO NPs were encapsulated with PLGA using an oil-in-water emulsion technique mediated by sonication [5]. Approximately 100 mg of PLGA was dissolved in 2 mL of dichloromethane (DCM) in a test tube. Once the polymer was fully dissolved, 50 mg of MnO NPs and 500 μL of a 2 mg/mL DCM solution of rhodamine 6G were added to the tube of the polymer/solvent mixture. The organic mixture was bath sonicated while being added dropwise to 4 mL of an aqueous 5% w/v solution of PVA while vortexing at high speed. The mixture was vortexed for an additional 10 s and then sonicated 3 X [15 s ON– 5 sec OFF] at 40% amplitude with a Qsonica Sonicator 125 Watts to create the single emulsion. Immediately after sonication, the emulsion was poured into 60 mL of an aqueous 0.3% w/v PVA solution, under rapid mixing on a stir plate. The PLGA MnO NPs were stirred for 3 hr to evaporate the DCM and were collected by centrifugation at 17,400 x g for 10 min. NPs were washed 3 times with deionized water, resuspended in deionized water, frozen overnight at -80°C, and dried on a lyophilizer for 3 days.

### Physical and chemical characterization of MnO NPs

To prepare samples for transmission electron microscopy (TEM), dried MnO NPs and PLGA MnO NPs were suspended in ethanol and deionized water, respectively, using bath sonication. After NP resuspension, 15 μL of the MnO NP mixture was dropped and air dried on 300 mesh copper PELCO® TEM grid support films of carbon type-B (Ted Pella, Inc.). Images were taken using a JEOL JEM-2100 transmission electron microscope at 200 kV for the MnO NPs and 120 kV for the PLGA MnO NPs.

X-ray diffraction patterns (XRD) of MnO NPs were obtained using a Panalytical X’Pert Pro X-ray diffractometer equipped with a Cu K-Alpha X-ray source operating at 45 kV and 40 mA in the Bragg-Brentano geometry. The spectra were collected over a 2θ range of 5° to 110° at a step size of 0.017° with a 1D silicon strip X-ray detector. The obtained XRD patterns were analyzed using the X’Pert HighScore Plus program. By comparing the XRD spectra of our synthesized MnO NPs with known spectra for MnO and Mn_3_O_4_, the program obtained an estimated composition for our samples.

Scanning electron microscopy (SEM) with energy dispersive x-ray spectroscopy (EDS) was performed to analyze the elemental composition of the MnO NP samples using a Hitachi Scanning Electron Microscope S4700 operated at 15 kV with the EDAX Team EDS System.

X-ray photoelectron spectroscopy (XPS) measurements were performed using a PHI VersaProbe 5000 Scanning X-Ray Photoelectron Spectrometer (ULVAC-PHI, Inc.) at room temperature and under vacuum greater than 1e^-6^ Pascal. All measurements were performed using a focused Al K-Alpha X-ray source at a photon energy of 1486 eV and power of 25 W with an X-ray spot size of 100 μm. The take-off angle of the photoelectron was set at 45^o^. Compositional survey scans were obtained using a pass energy of 117.4 eV and an energy step of 0.5 eV. High-resolution detailed scans of each element were acquired using a pass energy of 23.5 eV and an energy step of 0.1 eV. All XPS spectra were referenced to the C1s peak at a binding energy of 284.8 eV.

Fourier-transform infrared spectroscopy (FTIR) measurements on MnO NP samples, oleylamine, PLGA MnO NP samples, and PLGA were performed using a DIGILAB FTS 7000 FTIR spectrometer equipped with a GladiATR attenuated total reflectance (ATR) module from PIKE Technologies.

Size distribution of the PLGA MnO NPs was measured through dynamic light scattering (DLS) using a Nano Powder Sizer Malvern Instrument Zetasizer Nano ZS. Six milligrams of the PLGA MnO NPs were suspended in 10 mL of deionized water and bath sonicated prior to analysis.

### Mn^2+^ controlled release experiments

To evaluate Mn^2+^ release under different pH conditions, 10 mg of each MnO NP batch was suspended in 1 mL of PBS pH 7.4, 20 mM citrate buffer pH 6.5, or 20 mM citrate buffer pH 5 to simulate the pH of the blood, the tumor microenvironment, and cellular endosomes/lysosomes, respectively. The same three pH conditions were used to evaluate Mn^2+^ release from unencapsulated smaller MnO NPs (19 ± 6 nm) and unencapsulated smaller Mn_3_O_4_ NPs (17 ± 5 nm) to further observe the effect of size and chemical composition on release rate. Similarly, PLGA MnO NPs were suspended under the same conditions to assess Mn^2+^ release from hydrophilic NPs. Citrate buffers were made by adding anhydrous citric acid and sodium citrate dihydrate to deionized water. The MnO NP or PLGA MnO NP solutions were incubated in Eppendorf tubes under continuous slow rotation (6 rpm) to ensure gentle mixing over 24 hr at 37°C to simulate body temperature. At 1, 2, 4, 8 and 24 hr, the Eppendorf tubes were centrifuged at 17,400 x g for 10 min and the supernatants were collected and analyzed for released Mn^2+^ content by inductively coupled plasma-optical emission spectrometry (ICP-OES). The remaining pelleted MnO NPs or PLGA MnO NPs were resuspended in 1 mL of fresh buffer and placed back into the rotating incubator until the next time point was collected. The maximum amount of Mn^2+^ contained within each NP batch was calculated through measuring the total Mn^2+^ content of unencapsulated MnO NPs (10 mg) or PLGA MnO NPs (10 mg) fully dissolved in 150 μL of HCl trace metal grade using bath sonication. Mn^2+^ amounts were analyzed using Agilent 720 ICP-OES (1400 watts) with a plasma flow of 15.0 L/min, auxiliary flow of 1.50 L/min, and nebulizer flow of 0.75 L/min. Each sample was evaluated 5 times with a replicate and stabilization time of 10 and 15 s, respectively, and results were averaged. The % Mn^2+^ released at each time point was calculated using Eq 1 below. The Mn^2+^ cumulative release graph was created by adding together the % Mn^2+^ released from each of the previous time points.

$$\%{Mn}^{2+}\;released\;per\;time\;point=\frac{{mg\;of\;Mn}^{2+}\;released\;per\;time\;point}{mg\;of\;total\;{Mn}^{2+}\;in\;10\;mg\;of\;MnO\;NPs\;or\;PLGA\;MnO\;NPs}•100$$ (1)

PLGA MnO NP % loading capacity was calculated using the total Mn^2+^ content of unencapsulated MnO NPs (10 mg) and PLGA MnO NPs (10 mg) with Eq 2 below.

$$\%Loading\;capacity=\frac{{mg\;of\;total\;Mn}^{2+}}{10\;mg\;PLGA\;MnO\;NPs}•\frac{10\;mg\;of\;MnO\;NPs}{mg\;of\;total\;{Mn}^{2+}}•100$$ (2)

### MRI properties of NPs

Two different MRI experiments were performed. First, the r_1_ molar relaxivities of Mn^2+^ at pH 7.4 (PBS), pH 6.5 (20 mM citrate buffer), and pH 5 (20 mM citrate buffer) were determined. To measure r_1_, manganese(II) chloride tetrahydrate was dissolved in the three buffers to achieve Mn^2+^ concentrations of 0.182, 0.102, 0.058, 0.032, and 0.018 mM. MRI of the Mn^2+^ solutions was performed at 1.0 T on a Bruker ICON MRI. T_1_ measurements were generated by a RARE sequence using an echo time of 10.68 ms. A total of 10 repetition times (25.2, 50, 100, 200, 400, 800, 1,600, 3,200, 6,400, and 12,800 ms) were used to acquire images of the tubes. Using Matlab and the T_1_ longitudinal relaxation equation (Eq 3), T_1_ fitting was accomplished:

$$M_{z}=M_{o}•\left(1-e^{\frac{-t}{T_{1}}}\right)$$ (3)

where M_z_ is the longitudinal magnetization aligned along the z-axis at some time, t, and M_o_ is the magnetization at equilibrium. The r_1_ relaxivity for Mn^2+^ at pH 7.4, pH 6.5 and pH 5 was calculated using Eq 4:

$$\frac{1}{T_{1}}=\frac{1}{T_{1,0}}+[{Mn}^{2+}]•r_{1}$$ (4)

where 1/T_1_ is the measured relaxation rate, 1/T_1,0_ is the relaxation rate of the solvent only, and [Mn^2+^] is the concentration of Mn^2+^. The relaxivity is the slope of the linear fitted line when 1/T_1_−1/T_1,0_ is plotted versus [Mn^2+^].

Second, unencapsulated or PLGA encapsulated MnO NPs were suspended in pH 7.4, pH 6.5 and pH 5 as described for Mn^2+^ controlled release experiments. After 24 hours, the supernatant from 8 to 24 hr for the unencapsulated NPs was collected and diluted 100 fold. For PLGA encapsulated NPs, the supernatant from all time points were combined and diluted 100 fold. Longitudinal MRI properties of the collected supernatants containing released Mn^2+^ were measured at 1 T using the same MRI parameters as above. T_1_ values of the supernatants were measured using Eq 3. Additionally, Mn^2+^ concentrations were calculated from the measured T_1_ values and the r_1_ values for Mn^2+^ at each pH using Eq 4. Mn^2+^ concentrations measured by MRI were compared with Mn^2+^ concentrations measured by ICP-OES.

### Statistics

Approximately 25 to 35 TEM images and 800 to 900 MnO NPs were quantified per temperature condition using the line trace tool in ImageJ to measure the NP diameter. Each temperature condition contained 3 independent batches of synthesized MnO NPs. Statistical significance of mean NP diameters, Mn^2+^ controlled release, and MRI T_1_ values between groups were evaluated using the 2-tailed unpaired Student’s t-test with Bonferroni correction to account for multiple comparisons, where *p < 0.05 was defined as significant and **p < 0.01 was defined as highly significant. The polydispersity index (PDI) of MnO NPs for each temperature condition was calculated from the TEM images using Eq 5:

$$PDI={\left(\frac{\sigma }{d}\right)}^{2}$$ (5)

where σ is the standard deviation of the MnO NP diameters, and d is the mean diameter of MnO NPs.

## Results and discussion

**Fig 1:**
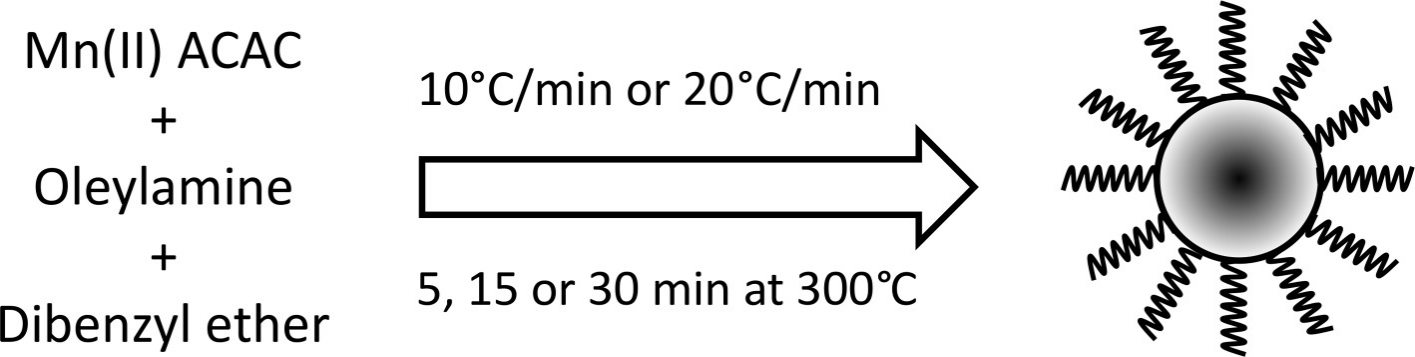
Thermal decomposition of Mn(II) ACAC was performed in oleylamine and dibenzyl ether at two temperature ramp rates and increasing aging times at 300°C to form MnO NPs coated with oleylamine.

MnO NPs were fabricated using a standard thermal decomposition reaction of Mn(II) ACAC dissolved in oleylamine and dibenzyl ether (Fig 1). Precise control over the temperature rise was achieved through programming a temperature controller (S1 Fig), which received real-time feedback through a thermocouple probe placed inside the reaction mixture. Two different temperature variables were studied, specifically heating rates and aging times, in attempts to achieve pure MnO NPs of smaller sizes. For simplicity, in the rest of the manuscript, we will refer to all synthesized manganese oxide NPs as MnO NPs, unless otherwise specified.

### Smaller NP diameters result from faster temperature ramp rate and shorter aging time

**Fig 2:**
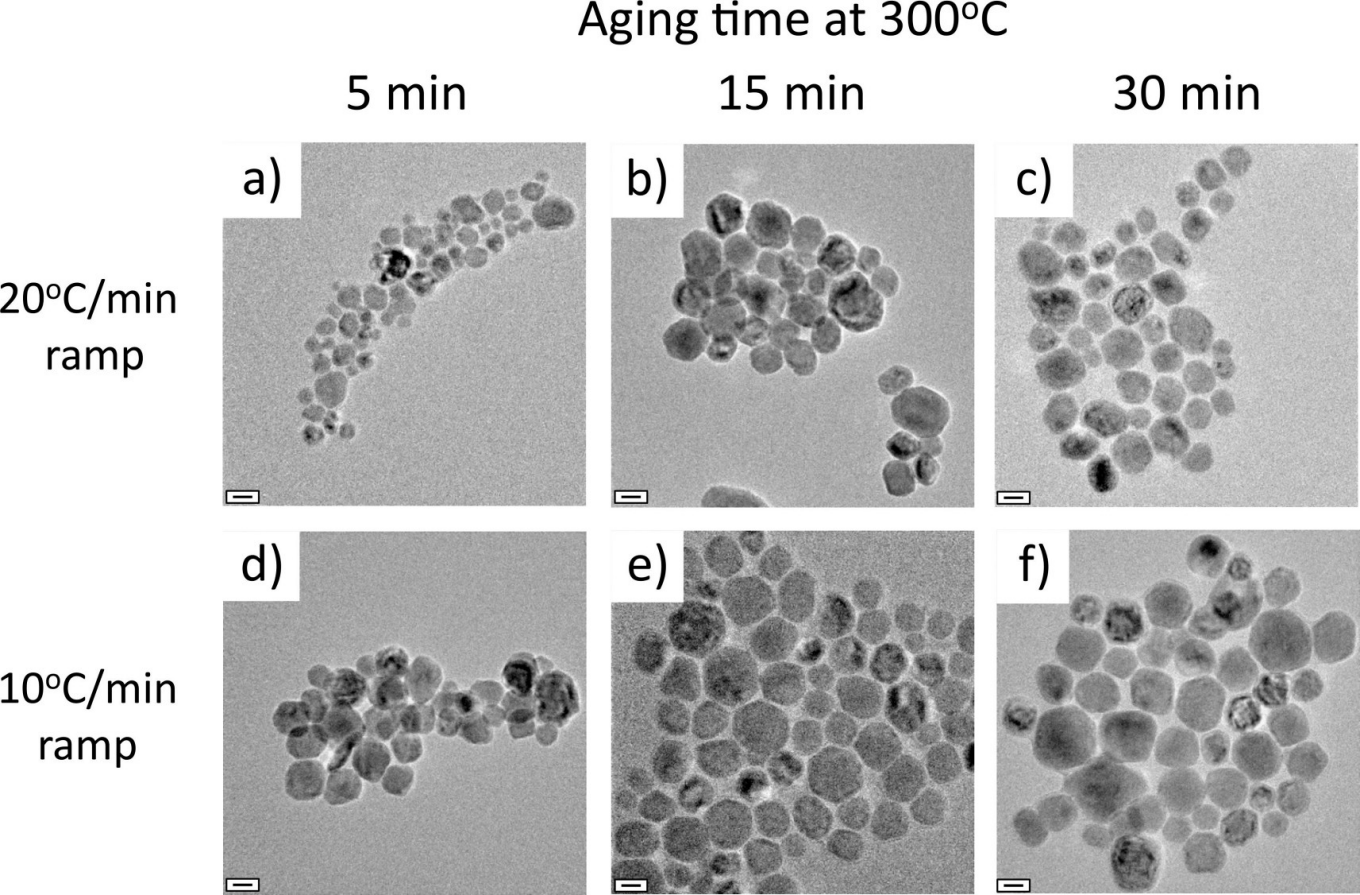
TEM images of MnO NPs generated from each of the 6 different temperature profiles: 20°C/min ramp with a) 5 min at 300°C, b) 15 min at 300°C, c) 30 min at 300°C, and 10°C/min ramp with d) 5 min at 300°C, e) 15 min at 300°C, and f) 30 min at 300°C. The MnO NPs have a rounded octagon shape, but some variation in size. Scale bars are 20 nm.

TEM was used to assess MnO NP size and a representative image from each temperature condition is shown in Fig 2. Our MnO NPs generally displayed a rounded octagon morphology similar to MnO NPs obtained by Nolis et al. [21] from heating manganese acetate and oleylamine to 250 and 300°C; however, their MnO NP diameters were much larger (100 nm and 70 nm, respectively, at 250°C and 300°C).

**Fig 3:**
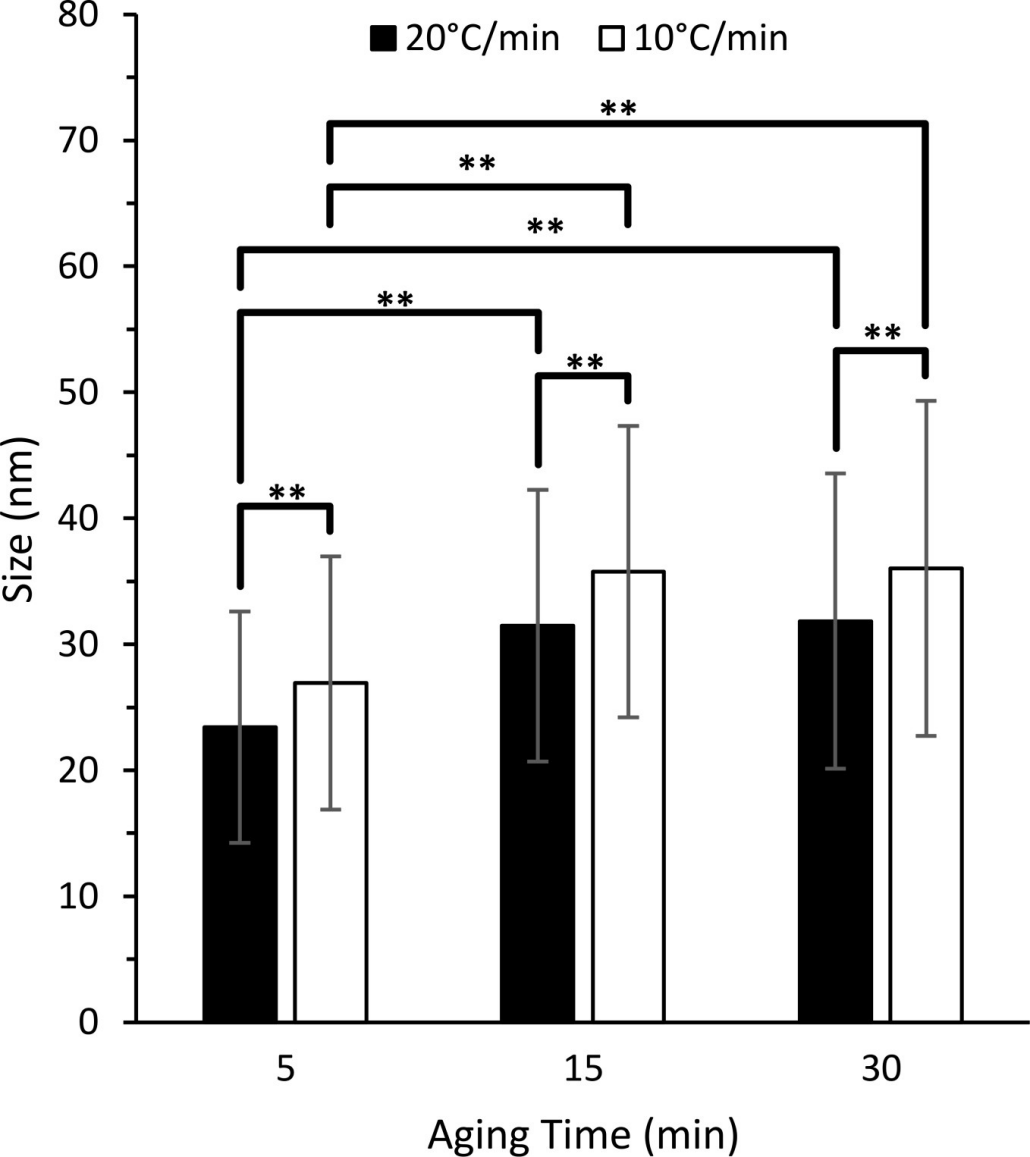
Average MnO NP diameters achieved with different ramping rates and aging times at 300°C. A faster ramping rate and a shorter aging time produced the smallest NPs. A total of 800–900 NPs were analyzed from TEM images per temperature condition. Error bars are average ± standard deviation. **p<0.01 was defined as highly significant.

As the ramping rate decreased and the aging time at 300°C increased, the average MnO NP diameter grew by a maximum of nearly 54%. When the fastest ramp (20°C/min) and shortest aging time (5 min) was used, the MnO NPs were the smallest, with an average diameter of 23 ± 9 nm. As the aging time was increased to 15 and 30 min, the average MnO NP size increased to 32 ± 11 nm and 32 ± 12 nm, respectively. When the ramp rate was decreased to 10°C/min, the average size of MnO NPs increased to 27 ± 10 nm, 36 ± 12 nm, and 36 ± 13 nm at 5, 15, and 30 min at 300°C, respectively. As shown in Fig 3, the average NP diameter was significantly different between ramping rates at all aging times. Significance was also achieved within both ramping rates when comparing aging times of 5 min to 15 min and 5 min to 30 min. Despite the high standard deviation of NP size, significance was achieved due to the large sample size analyzed (800–900 NPs per temperature condition). PDI values for MnO NPs from each temperature condition were calculated and found to be ≤ 0.15 (S1 Table). It is important to note that after 15 min at 300°C, the MnO NP size stabilized in both ramping conditions.

The increase in MnO NP size with a slower temperature ramp and an increase in aging time at 300°C was likely due to a longer total reaction time, leading to more opportunity for NP growth and coalescence (S1 Table). Histograms comparing the size distributions at the two different temperature ramps are shown in S2 Fig. Chen et al. [28] also observed a rise in NP growth with increasing aging times at 310°C, which was associated with two distinct growth patterns: only minimal increases in NP size were observed from 3 to 30 min at 310°C, whereas a much larger increase in NP size was achieved from 100 to 285 min at 310°C.

Our MnO NPs tended to exhibit a variation in size, likely due to several factors. First, MnO NP growth could follow an Ostwald ripening process, whereby smaller NPs begin to dissolve and add onto larger ones to cause polydispersity [28]. Second, a subset of smaller MnO NPs could coalesce or join together into larger NPs as the reaction proceeds to also lead to size variation [30–32]. Third, the concentration of oleylamine has been shown to contribute to NP size distribution. When a lower stabilizer concentration is used, the NPs do not have enough capping, which can allow for their aggregation [33].

### Mn_3_O_4_ is incompletely reduced to MnO by faster temperature ramp rate and shorter aging time

**Fig 4:**
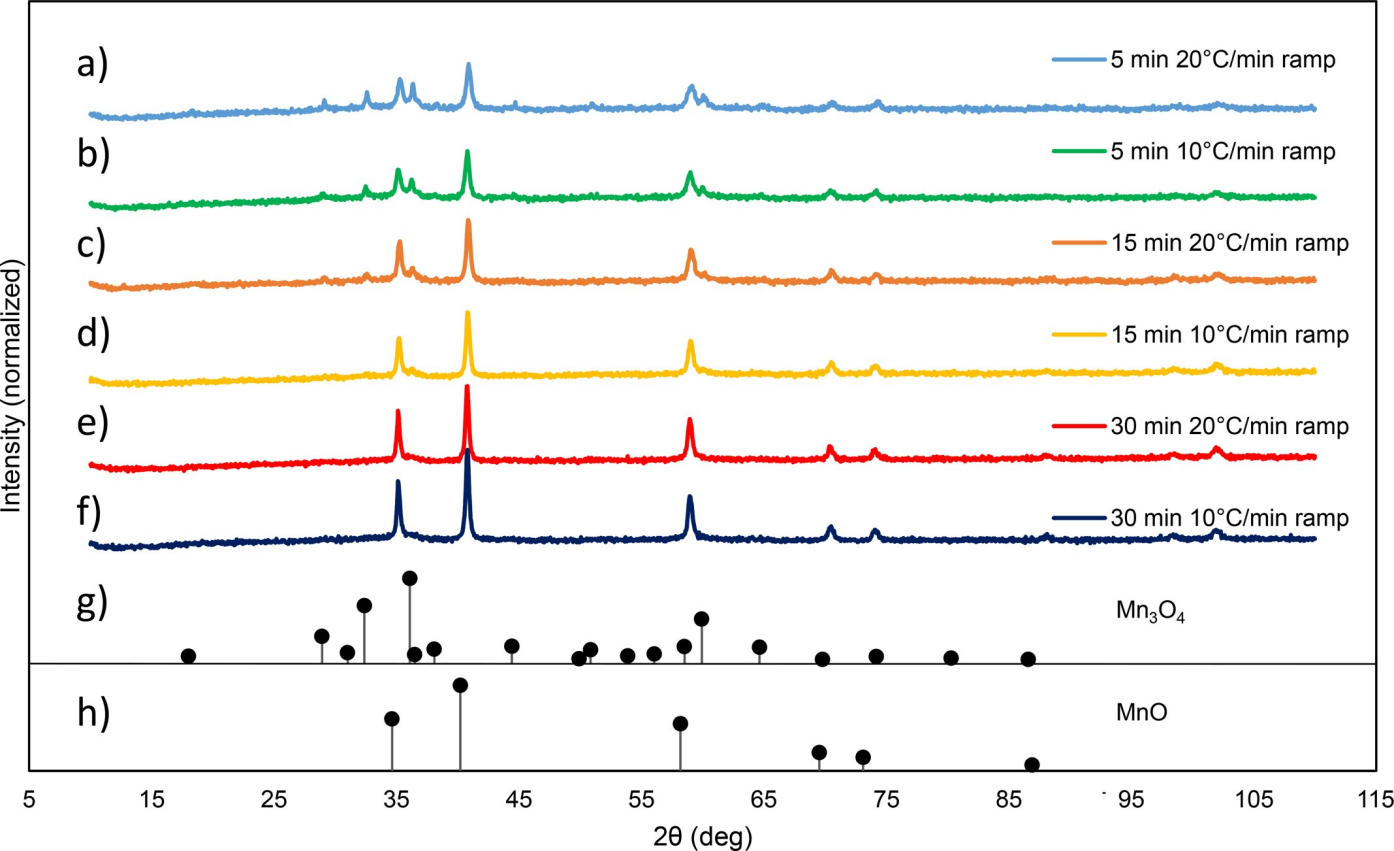
XRD spectra of Mn_3_O_4_/MnO NP mixture or MnO NPs generated with the following temperature profiles: a) 5 min at 300°C with 20°C/min ramp, b) 5 min at 300°C with 10°C/min ramp, c) 15 min at 300°C with 20°C/min ramp, d) 15 min at 300°C with 10°C/min ramp, e) 30 min at 300°C with 20°C/min ramp, and f) 30 min at 300°C with 10°C/min ramp. The standard diffraction peaks for known g) Mn_3_O_4_ and h) MnO are shown from X’Pert HighScore. Upon comparison with the standard spectra, a-c) shows Mn_3_O_4_/MnO NP mixtures, whereas d-f) shows MnO NPs.

Remarkably, the MnO NP size was not the only characteristic affected when the temperature profile was changed. XRD was used to evaluate MnO NP crystal structure and bulk composition. Fig 4A–4F shows the XRD spectra of each NP for all 6 temperature conditions, while Fig 4G and 4H show the characteristic XRD peaks of Mn_3_O_4_ and MnO, respectively. All synthesized NPs (Fig 4A–4F) clearly display the 5 highest characteristic peaks for MnO (Fig 4H), whereas the first three spectra (Fig 4A–4C) also contain the 3 highest characteristic peaks of Mn_3_O_4_ (Fig 4G). Therefore, the top 3 temperature profiles with the shortest total reaction times resulted in a mixture of Mn_3_O_4_ and MnO NPs and the bottom 3 temperature profiles with longer total reaction times led to a more pure MnO NP formulation. Table 1 displays the estimated percent NP composition that X’Pert HighScore Plus calculated for each temperature profile based on its database of known compounds. As the overall temperature reaction times were increased, the MnO percentage composition increased and Mn_3_O_4_ percentage composition decreased. We hypothesize that when less time was applied into the synthesis, the reaction did not have enough thermal energy to occur completely, obtaining a mixture of MnO and Mn_3_O_4_.

**Table 1:** MnO and Mn_3_O_4_ composition (%) for NP trials based on X’Pert HighScore database.

Temperature Condition (aging time, ramp rate)	Composition (%)	
	MnO	Mn_3_O_4_
5 min, 20°C/min ramp Trial #1	58	42
5 min, 20°C/min ramp Trial #2	40	60
5 min, 20°C/min ramp Trial #3	45	55
5 min, 10°C/min ramp Trial #1	68	32
5 min, 10°C/min ramp Trial #2	68	32
5 min, 10°C/min ramp Trial #3	63	37
15 min, 20°C/min ramp Trial #1	83	17
15 min, 20°C/min ramp Trial #2	66	34
15 min, 20°C/min ramp Trial #3	88	12
15 min, 10°C/min ramp Trial #1	89	11
15 min, 10°C/min ramp Trial #2	84	16
15 min, 10°C/min ramp Trial #3	85	19
30 min, 20°C/min ramp Trial #1	90	10
30 min, 20°C/min ramp Trial #2	87	13
30 min, 20°C/min ramp Trial #3	92	8
30 min, 10°C/min ramp Trial #1	86	14
30 min, 10°C/min ramp Trial #2	88	12
30 min, 10°C/min ramp Trial #3	91	9

To our knowledge, our study is the first to show that the ramping rate and aging time at 300°C impact the composition of the synthesized NPs. Mn_3_O_4_ or MnO/Mn_3_O_4_ NP mixtures were previously observed by Nolis et al. [21] and Seo et al. [22], but the aging temperature used was much lower between 150–200°C. Our study reveals that MnO NPs still contain some Mn_3_O_4_ composition even at 300°C when applying faster ramp rates and shorter aging times. Based on our results and the literature, we hypothesize that the formation of MnO NPs is initiated by first forming Mn_3_O_4_ NPs at lower temperatures (150–200°C) during thermal decomposition of Mn(II) ACAC. As the reaction time and temperature is increased, the Mn_3_O_4_ NPs begin to be reduced to MnO NPs through an endothermic reaction: Mn_3_O_4_ → 3MnO + ½O_2_ [34]. Shorter aging times at 300°C do not allow for complete conversion of Mn_3_O_4_ to MnO, and lead to a mixed MnO/Mn_3_O_4_ composition. Longer aging times provide more thermal energy needed to obtain a full reduction to a pure MnO composition. Once again, for simplicity, in the rest of the manuscript, we will refer to all synthesized manganese oxide NPs as MnO NPs.

### MnO NP surfaces are coated with Mn_3_O_4_ and oleylamine

**Fig 5:**
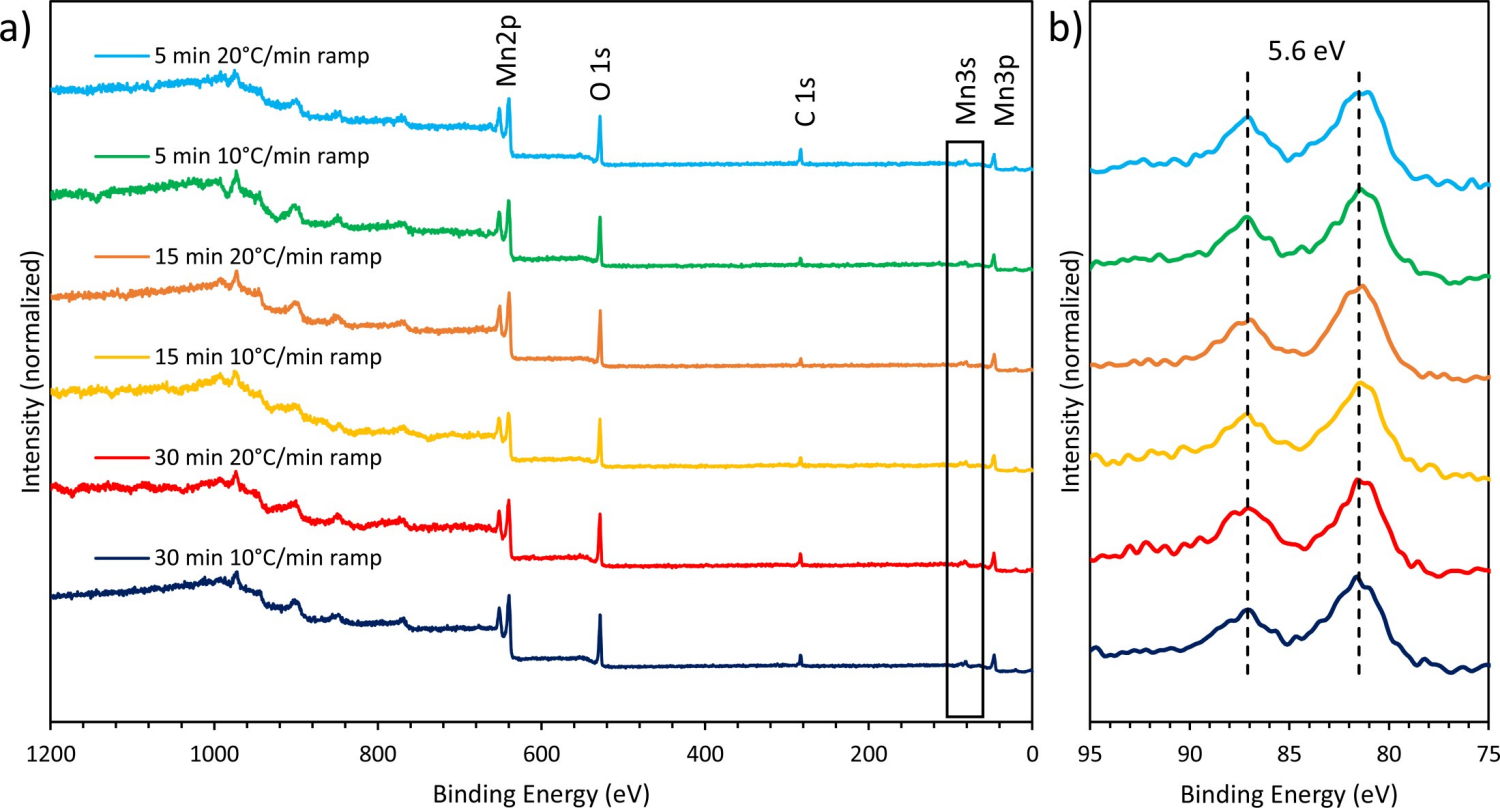
XPS spectra of MnO NP samples for each temperature profile showing the a) whole spectral region and b) the Mn3s region. The whole spectral region indicates the presence of manganese, oxygen, and carbon in the NP samples, while the Mn3s region shows peak splitting characteristic of surface oxidation to Mn_3_O_4_ (Mn^2+^/Mn^3+^ oxidation states).

To complement the bulk analysis of XRD, SEM/EDS, XPS and FTIR were used to assess the surface chemistry of MnO NPs formed with different temperature profiles. SEM/EDS and XPS confirmed the elemental composition of our NP samples to be mainly manganese and oxygen (S3 Fig and Fig 5A, respectively). The magnitude of the Mn3s peak splitting (Fig 5B) can be used to identify the oxidation state of surface bound manganese. A ΔE of 6.1eV indicates MnO (Mn^2+^), while a ΔE of ≥ 5.4 eV indicates Mn_2_O_3_ (Mn^3+^) [35]. Previous literature has shown that since Mn_3_O_4_ is a mixture of Mn^2+^ and Mn^3+^ oxidation states, the Mn3s peak splitting has an intermediate ΔE of 5.6 eV [36]. Fig 5B shows that all NP samples, regardless of the temperature profile, show the characteristic peak splitting of Mn_3_O_4_. XPS results demonstrate that the surface of the NPs oxidizes after synthesis in the presence of air to form a coating of Mn_3_O_4_, consistent with what others have found [36]. Together, XRD and XPS show that reaction conditions affect the overall bulk composition of the synthesized NPs (Mn_3_O_4_/MnO versus MnO), but that all NPs are oxidized to include a layer of Mn_3_O_4_ on the surface.

**Fig 6:**
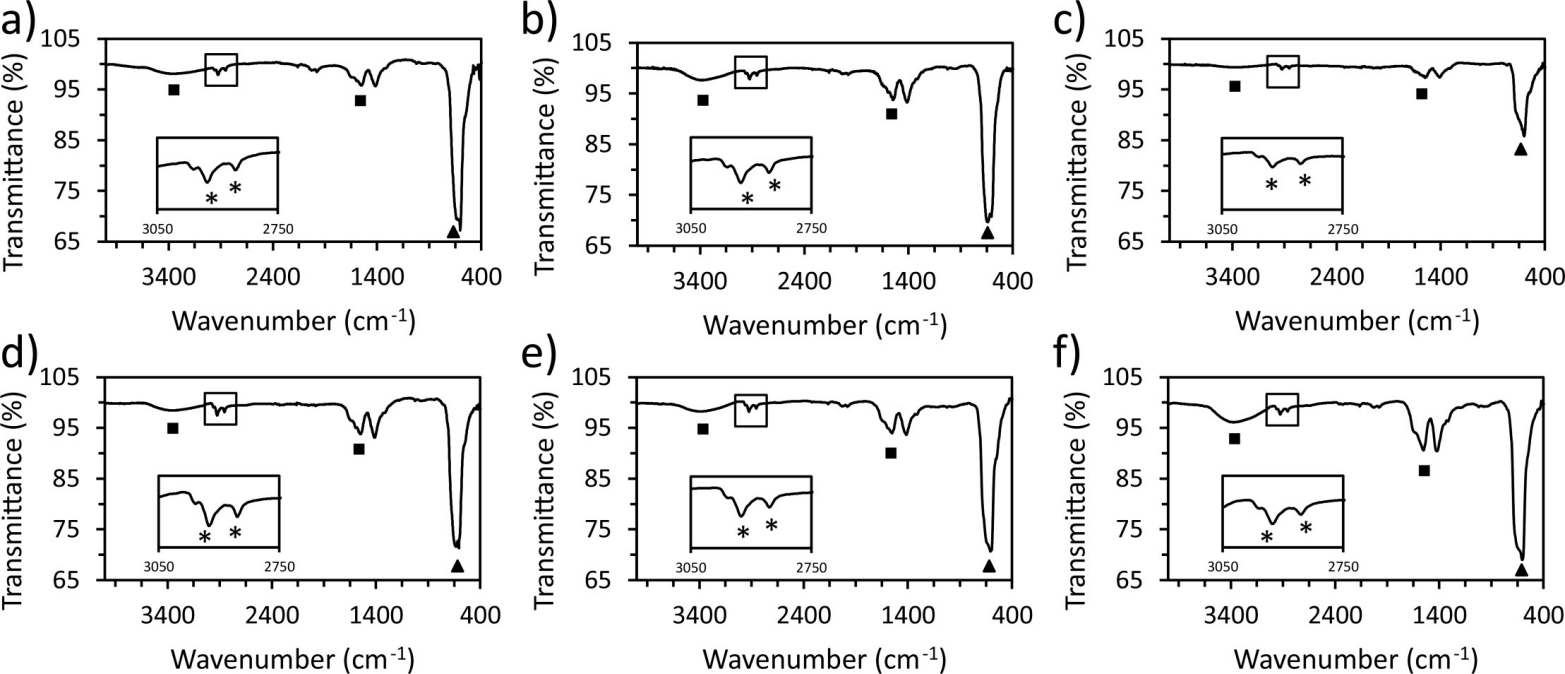
FTIR spectra of the following temperature profiles: 20°C/min ramp with a) 5 min at 300°C, b) 15 min at 300°C, c) 30 min at 300°C, and 10°C/min ramp with d) 5 min at 300°C, e) 15 min at 300°C, and f) 30 min at 300°C. Asterisks represent oleyl groups, squares correspond to amine groups, and triangles show the vibration of Mn-O and Mn-O-Mn bonds. The oleyl group spectral regions are enlarged in the boxed insets to resolve the two distinct peaks.

The NP surface chemistry was further studied with FTIR to corroborate hydrophobic capping with oleylamine. Fig 6 presents the FTIR spectra of each NP for all 6 temperature conditions. All NP samples show the characteristic modes of oleyl groups: peaks around 2850–2854 and 2918–2926 cm^-1^ (marked by asterisks) due to the symmetric and asymmetric CH_2_ stretching modes, respectively [37]. Additionally, the peaks around 1593 cm^-1^ and 3300 cm^-1^ (marked by squares) are attributed to the NH_2_ bending vibration, and the symmetric and asymmetric stretching vibration of the amine group (NH_2_), respectively [38]. MnO NP FTIR spectra had similar peaks to those present in the oleylamine only spectra (S4 Fig). These peaks consolidate NP capping formed by oleylamine, which is consistent with the literature when synthesizing metal oxide NPs [29, 39]. Lastly, peaks around 600 cm^-1^ (marked by a triangle) correspond to the vibration of Mn-O and Mn-O-Mn bonds, confirming the chemistry found through XRD, SEM/EDS and XPS [40].

### Mn^2+^ release rate from MnO NPs is maximum at pH 5 and unaffected by synthesis conditions

**Fig 7:**
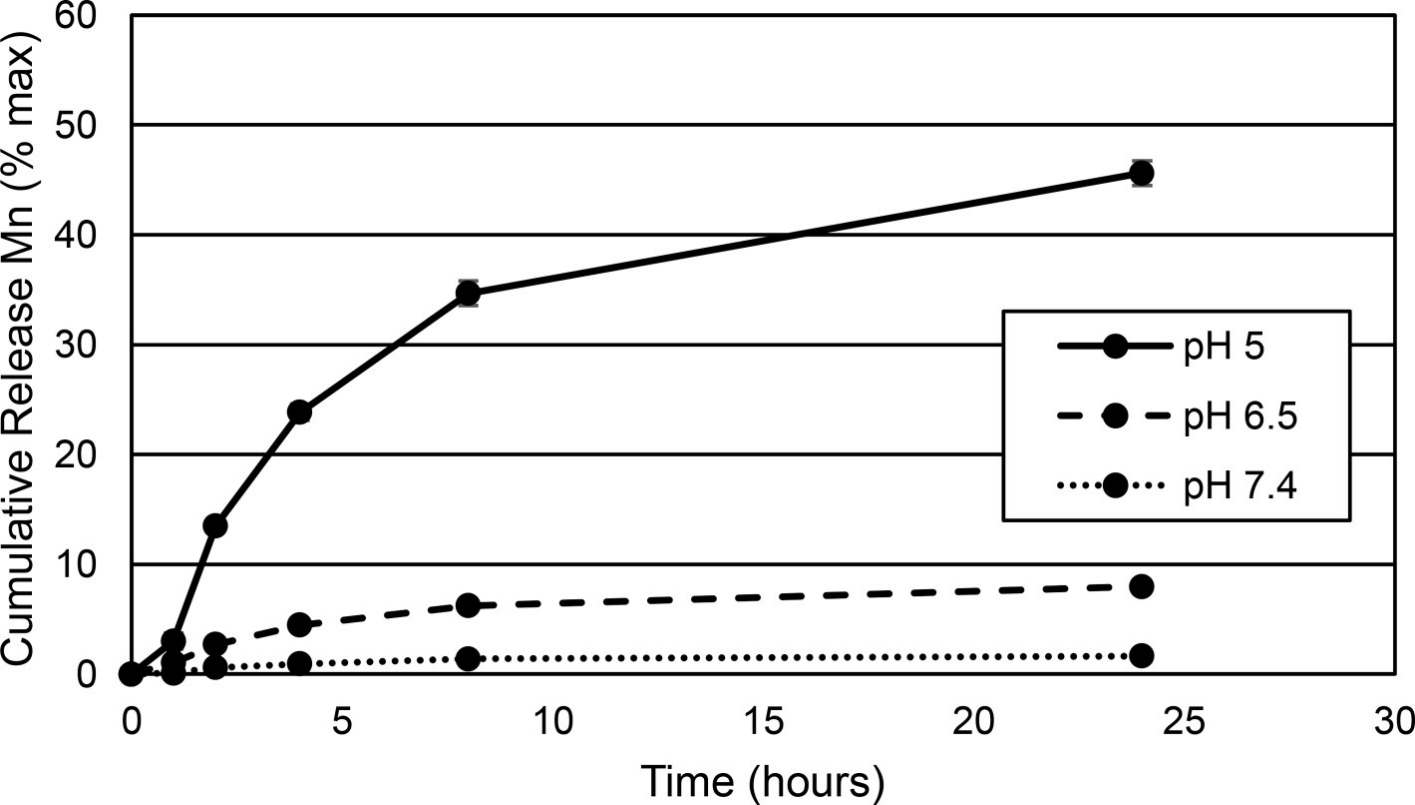
**Cumulative release of Mn**^**2+**^ **from MnO NPs over 24 hr after incubation in PBS pH 7.4 (dotted line), 20 mM citrate buffer pH 6.5 (dashed line), and 20 mM citrate buffer pH 5 (solid line).** Mn^2+^ release increased with a decrease in pH. The controlled release curve is shown for the MnO NPs generated with 30 min at 300°C and a 20°C/min ramp. Time points are shown for 1, 2, 4, 8 and 24 hr. Error bars show mean ± standard deviation.

As mentioned before, size reduction of MnO NPs is important to increase the surface area to volume ratio to generate a higher dissolution rate of MnO to Mn^2+^ in acidic media to create a greater T_1_ MRI signal. The controlled release profile of Mn^2+^ from MnO NPs was tested over time in 3 different pH conditions: pH 7.4 to mimic the normal physiological pH of the blood, pH 6.5 to mimic the slightly acidic extracellular pH in cancerous tumors due to increased lactic acid production, and pH 5 to mimic the acidic pH of endosomes and lysosomes inside cells. It is well known that following cell uptake, metallic NPs are shuttled to endosomes inside cells [41]. Fig 7 shows a representative Mn^2+^ controlled release curve for MnO NPs formed from the 20°C/min ramp aged at 300°C for 30 min. Similar to what we have shown previously [5], MnO dissociation into Mn^2+^ at physiological pH 7.4 was extremely minimal (faint dotted line in Fig 7), meaning that no MRI signal enhancement would be produced in the blood. At pH 6.5, only ~9% (~0.5 mg) of the total manganese content was released as Mn^2+^ after 24 hr (dashed line in Fig 7), which will likely result in a very weak enhancement of MRI signal if the MnO NPs remain in the extracellular space of cancerous tumors. Consistent with our previous findings [5], pH 5 showed the most robust release of Mn^2+^ over 24 hr of ~46% (~2.9 mg) (solid black line in Fig 7). The other MnO NP formulations (S5 Fig) showed similar controlled release curves with no significant differences between Mn_3_O_4_/MnO NP mixtures and MnO only. The total Mn^2+^ amount released at each temperature condition and pH are shown in S2 Table.

The similarity of Mn^2+^ release from all NP formulations in acidic media was surprising, as Godunov et al. [42] have shown that Mn_3_O_4_ dissolves incompletely in dilute acidic conditions due to the formation of Mn^2+^ ions as well as MnO_2_, whereas MnO dissolves completely. Their study also demonstrated that MnO dissolves at a faster rate than Mn_3_O_4_ in concentrated acidic solutions, but both compounds completely dissociate [42]. The similarity of our Mn^2+^ controlled release curves between NP formulations could indicate that the slower anticipated Mn^2+^ release from MnO/Mn_3_O_4_ NP mixtures could be counteracted by their smaller NP diameter with increased surface area. Similarly, MnO NPs would be expected to exhibit enhanced Mn^2+^ production, but had larger diameters, which could slow their release.

### Small diameter MnO NPs have maximum Mn^2+^ release and MRI signal enhancement

**Fig 8:**
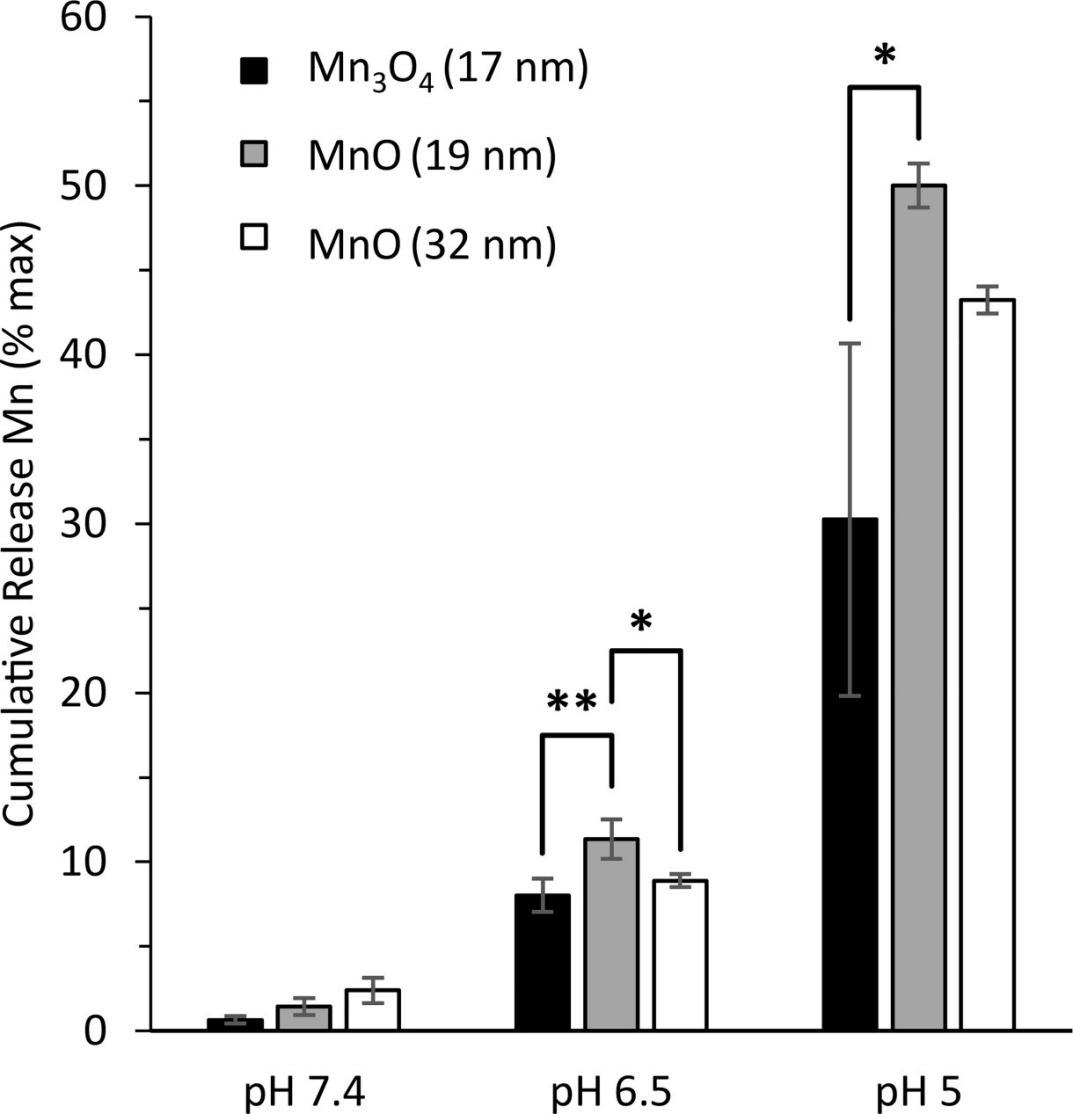
Average cumulative release of Mn^2+^ from 17 nm Mn_3_O_4_, 19 nm MnO, and 32 nm MnO NPs after 24 hr of incubation in PBS pH 7.4, 20 mM citrate buffer pH 6.5, and 20 mM citrate buffer pH 5. Mn^2+^ release was highest from small MnO NPs and lowest from small Mn_3_O_4_ NPs at pH 5 and 6.5. Three different batches of each type of NPs were analyzed during controlled release. Error bars are average ± standard deviation.* p<0.05 and **p<0.01 were defined as significant and highly significant, respectively.

To further understand how NP size and chemical composition affected Mn^2+^ controlled release and T_1_ MRI properties, we compared the synthesized optimal MnO NPs above (32 nm ± 12 nm) with smaller MnO NPs (19 ± 6 nm) and smaller Mn_3_O_4_ NPs (17 ± 5 nm). TEM images and XRD spectra of the small MnO and small Mn_3_O_4_ NPs are shown in S6 Fig. When compared to large MnO NPs, small MnO NPs released ~7% more Mn^2+^ after 24 hours at pH 5 likely due to their increased surface area to volume ratio; however, the increased Mn^2+^ production from small MnO NPs was not statistically significant (Fig 8). Chemical composition of NPs had a much larger impact on Mn^2+^ release. Small Mn_3_O_4_ NPs released significantly less Mn^2+^ (~20% reduction) than small MnO NPs (Fig 8). Similar trends were observed at pH 6.5.

Next, the impact of NP size and chemical composition on T_1_ MRI signal enhancement was assessed to determine which formulation would be most favorable for MRI applications. The smallest MnO NPs were the most efficient MRI contrast agents by producing the lowest T_1_ value at all pH levels (Table 2). This is not surprising, as small MnO NPs released the greatest amount of Mn^2+^ after 24 hours at pH 5 and 6.5 (Fig 8). Large MnO NPs were slightly less efficient with a ~20% increase in T_1_ compared to small MnO NPs. Small Mn_3_O_4_ NPs were the least effective MRI contrast agents, with a ~42% increase in T_1_ compared to small MnO NPs that was statistically significant (Table 2). Similar to small MnO NPs, the obtained T_1_ values for large MnO NPs and small Mn_3_O_4_ NPs also mirrored the trends seen in the Mn^2+^ controlled release experiments at pH 6.5 and 5 (Fig 8). Therefore, for MRI applications, it would be favorable to utilize MnO NPs rather than Mn_3_O_4_ due to the generation of a greater concentration of Mn^2+^ ions which produces a larger signal enhancement on T_1_ MRI (S7 Fig). Furthermore, the MRI data confirmed the results from ICP-EOS when using the Mn^2+^ calibration curves to calculate the Mn^2+^ concentration in the solutions (S7 Fig). Since the calibration curves only contained diluted Mn^2+^ ions and the calculated Mn concentration from MRI and ICP-OES were similar at pH 6.5 and 5 (S3 Table), it is likely that Mn^3+^ ions minimally contributed to the MRI signal and Mn^2+^ ions were the dominant species responsible for the MRI signal increase. According to Gale et al. [43], chelated Mn^2+^ has a 6.6 fold higher r_1_ relaxivity compared to chelated Mn^3+^ at 1.4 T, which supports that our main MRI signal likely originates from Mn^2+^.

**Table 2:** MRI T_1_ values of supernatants collected between 8 and 24 hours after NP incubation at pH 7.4, 6.5 and 5.

Nanoparticle Type (diameter)	T_1_ (ms)		
	pH 7.4	pH 6.5	pH 5
Mn_3_O_4_ (17 nm)	2,510 ±346	1,973 ±254	915 ±105*
MnO (19 nm)	2,350 ±174	1,647 ±32	646 ±16
MnO (32 nm)	2,449 ±286	1,755 ±202	774 ±89

*p<0.05 for Mn_3_O_4_ (17 nm) versus MnO (19 nm) at pH 5

### MnO NPs encapsulated in PLGA retain maximum Mn^2+^ release and MRI signal enhancement compared to PLGA Mn_3_O_4_ NPs

The MnO NPs synthesized herein are hydrophobic initially and capped with oleylamine (Fig 6). The hydrophobicity of MnO NPs in our study may provide a limitation to the assessment of NP dissolution kinetics. Therefore, hydrophilic NPs were fabricated by encapsulating large MnO NPs (32 nm), small MnO NPs (19 nm) and small Mn_3_O_4_ NPs (17 nm) within PLGA, a clinically approved biocompatible and biodegradable polymer, to confirm the trend observed with hydrophobic NPs. TEM showed dark metal oxide nanocrystals trapped within the polymeric NPs (S8 Fig) while FTIR confirmed successful surface coating with PLGA through matching characteristic FTIR spectral peaks of PLGA MnO NPs with PLGA only (circles shown in S9 and S10 Figs). PLGA MnO NPs had ~30% loading capacity (S4 Table) and an average diameter between 220 and 255 nm based on DLS analysis (S11 Fig).

**Fig 9:**
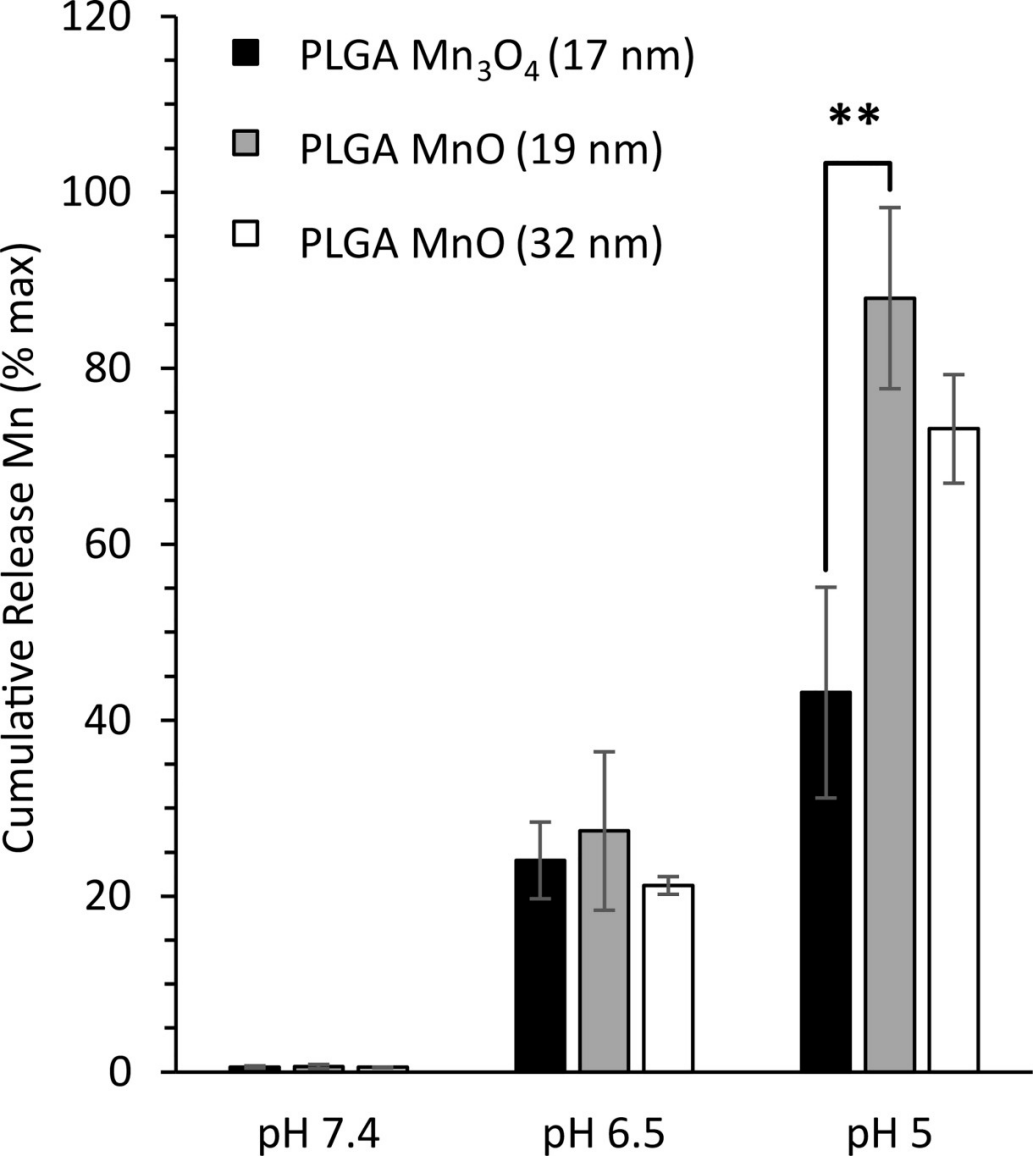
Average cumulative release of Mn^2+^ from PLGA Mn_3_O_4_ (17 nm), PLGA MnO (19 nm), and PLGA MnO (32 nm) NPs after 24 hr of incubation at pH 7.4, pH 6.5, and pH 5. Mn^2+^ release was significantly greater from PLGA MnO NPs compared to PLGA Mn_3_O_4_ NPs at pH 5. Three different batches of each type of NPs were analyzed during controlled release. Error bars are average ± standard deviation. **p<0.01 was defined as highly significant.

To test the effects of polymer coating on Mn^2+^ generation and MRI signal, the same controlled release experiment was performed on PLGA MnO NPs, and the supernatants were analyzed with ICP-OES and MRI. PLGA MnO (19 nm) NPs had the highest Mn^2+^ release after 24 hr at pH 5; approximately 90% of the encapsulated MnO NPs dissociated to Mn^2+^ compared to only ~45% of encapsulated Mn_3_O_4_ NPs (Fig 9). All three PLGA NPs had ~25% cumulative release rates at pH 6.5 after 24 hours, with negligible release at PBS pH 7.4 (Fig 9). As can be seen by comparing Figs 8 and 9, polymer encapsulation did not significantly impact the overall trends in Mn^2+^ controlled release. MnO retained its ability to produce Mn^2+^ at a much faster rate compared to Mn_3_O_4_ regardless of the surface groups.

PLGA MnO (19 nm) NPs had a significantly lower T_1_ value compared to PLGA Mn_3_O_4_ (17 nm) NPs after 24 hours at pH 5 (Table 3), which is consistent with the enhanced Mn^2+^ generation of MnO shown by ICP-OES. Both PLGA MnO NP formulations had comparable T_1_ values at all pHs (Table 3 and S12 Fig), which shows that unencapsulated MnO NP size does not have a significant impact on Mn^2+^ release or MRI signal once NPs are encapsulated within a polymer. Since a different number of metal oxide NPs can be encapsulated within each individual PLGA NP, variability in loading within each sample could have contributed to a loss of MRI trends between PLGA MnO (19 nm) NPs and PLGA MnO (32 nm) NPs. Altogether, our results support the use of MnO over Mn_3_O_4_ due to higher Mn^2+^ generation and MRI signal with and without PLGA encapsulation.

**Table 3:** MRI T_1_ values of supernatants collected 24 hours after PLGA NP incubation at pH 7.4, 6.5 and 5.

Nanoparticle Type (metal oxide diameter)	T_1_ (ms)		
	pH 7.4	pH 6.5	pH 5
PLGA Mn_3_O_4_ (17 nm)	2,340.6 ± 57.9	1,749.8 ± 81.0	1,404.4 ± 220.7**
PLGA MnO (19 nm)	2,263.7 ± 28.9	1,597.3 ± 52.1	854.8 ± 103.6
PLGA MnO (32 nm)	2,043.1 ± 237.2	1,605.2 ± 78.2	830.1 ± 49.1

**p<0.01 for PLGA Mn_3_O_4_ (17 nm) versus PLGA MnO (19 nm) at pH 5

In addition to utilizing MnO over Mn_3_O_4_, another strategy to maximize MRI signal from MnO NPs *in vitro* and *in vivo* would be to employ NP targeting to enhance uptake into tumor cells to take advantage of the low acidic conditions of endosomes and lysosomes to aid in increased Mn^2+^ generation. Several receptors are overexpressed on tumor cells depending on the cancer type such as the folate receptor, epidermal growth factor receptor (EGFR), human epidermal growth factor receptor 2 (HER2), the transferrin receptor, and the mucin-1 (MUC-1) receptor [44], among others. NPs can be conjugated with either antibodies or peptides designed to bind specifically to these receptors to enable targeting. Small targeting peptides provide several advantages over antibody targeting including reduced production cost, low molecular weight and reduced immunogenicity [45].

Besides PLGA encapsulation, NPs can be made hydrophilic through ligand exchange [35] or lipid capping [46]. Further NP modifications to enhance functionality include adding stealth polymers to the surface such as polyethylene glycol (PEG) [47] to extend blood circulation times to promote NP accumulation in tumors as well as adding chemotherapeutic drugs or microRNA to develop theranostic systems to track drug delivery to tumors. If PEG is used, targeting moieties should be added to the end of the PEG chains, as targeting agents attached to the NP surface would experience steric hindrance by long PEG chains and be inaccessible to bind with tumor cell receptors [48]. Although PEG can greatly enhance blood circulation times and NP accumulation in tumors, it can decrease NP uptake into tumor cells [49]. As an alternative approach, cleavable PEG chains can be utilized, which can be designed for cleavage at low tumor pH or by enzymes overexpressed at the cancer site [50]. Phospholipid versus polymer encapsulation techniques have different advantages. Phospholipid coating will minimally add to the overall NP size and facilitate synthesis of small NPs, as the hydrophobic lipid tail will associate with the hydrophobic NP surface and the hydrophilic head will point out towards the aqueous media [46, 51, 52]. Phospholipids conjugated to fluorescent dyes, polymers, and different reactive functional groups (e.g. free acid, amine, alkene, azido, etc.) are readily available from commercial sources such as Avanti Polar Lipids. When utilizing phospholipids, it is important to use long chain saturated lipids with a phase transition temperature >37°C to assure better stability and to purchase reactive functional groups and polymers attached to the lipid head groups to ensure these moieties are facing out towards the aqueous media. Polymeric encapsulation typically produces larger NPs, but is very customizable; fluorescent dyes, metal oxide NPs, and drug can all be added during the synthesis phase for simultaneous encapsulation [5, 53, 54]. Release rate of the contents can be controlled through changing the polymer composition.

**Fig 10:**
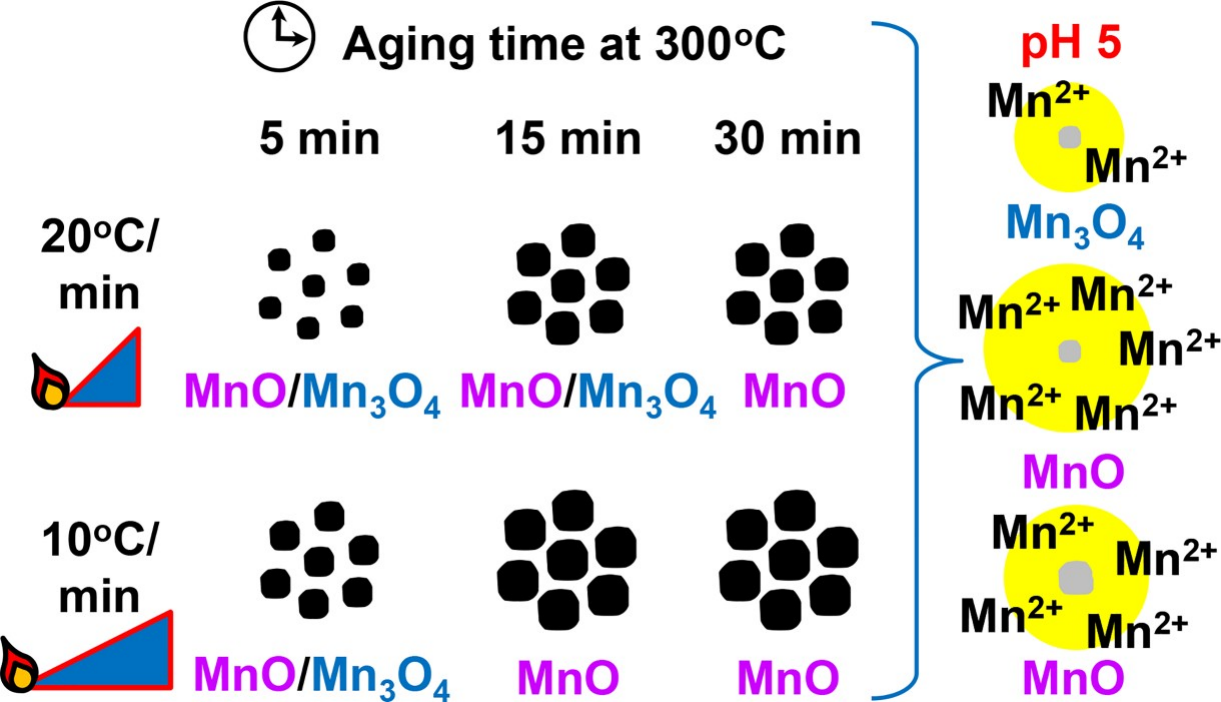
Schematic presentation illustrating how size and composition of MnO NPs can be fine-tuned by altering temperature ramping rate and aging time. A shorter aging time and faster ramp rate produced smaller NPs with a mixed composition of MnO/Mn_3_O_4_. Larger NPs comprised of MnO only were synthesized by extending the aging time and using a slower temperature ramp. Mn^2+^ production was highest at pH 5, mimicking cell endosomes. Unencapsulated small MnO NPs released the greatest amount of Mn^2+^ and had the highest MRI signal enhancement (yellow) compared to unencapsulated large MnO NPs and small Mn_3_O_4_ NPs. Although there was no significant difference between large and small MnO NPs after PLGA encapsulation, both PLGA MnO NP formulations released significantly more Mn^2+^ compared to PLGA Mn_3_O_4_ NPs and generated much higher T_1_ signal enhancement (not shown).

It will also be important to evaluate potential Mn toxicity in the consideration of adopting MnO NPs for MRI of tumors. Mn toxicity is thought to arise from the release of free Mn^2+^ ions, as Mn^2+^ mimics Ca^2+^ and can enter neurons and muscles. The ability of Mn^2+^ to travel down neurons has been used for manganese enhanced MRI (MEMRI) in animals to visualize neuronal activity. Bock et al. [55] have shown that MEMRI in rats had no adverse effects using 30 mg/kg of free Mn^2+^, injected every 2 days for 12 days, totaling 180 mg/kg Mn^2+^. MnO NPs should be better tolerated *in vivo*, as they carry MnO, not free Mn^2+^ directly. Through incorporating specific NP targeting to tumor cells and confining Mn^2+^ release to low pH tumor endosomes (Figs 7–9), the systemic dose of free Mn^2+^ should be minimized. Nonetheless, it will be necessary to thoroughly evaluate MnO NP hepatic, cardiac, and sensorimotor toxicity *in vivo* over time in tumor bearing animals to assess any off-target effects. The key results from our study are summarized below in Fig 10.

## Conclusions

In conclusion, we are the first to demonstrate that modification of the temperature ramp rate and aging time at 300°C can be used to fine-tune both the diameter and composition of MnO NPs (Fig 10). The fastest ramp and shortest aging time produced the smallest NP size through limiting the overall reaction time and NP growth; however, a mixture of Mn_3_O_4_ and MnO NPs was obtained with shorter aging times due to the incomplete reduction of Mn_3_O_4_ to MnO. To achieve pure MnO, which is most desirable for MRI applications, longer aging times at 300°C were needed, but MnO NP size increased as well. In our study, the 20°C/min temperature ramp with a 30 minute aging time at 300°C was the most ideal temperature condition to form the smallest pure MnO NPs. XPS and FTIR confirmed NP surface oxidation to Mn_3_O_4_ and oleylamine capping, respectively. Remarkably, ramping rate and aging time had a negligible effect on the Mn^2+^ release rate, indicating that NP size and composition characteristics could be counteracting each other, as MnO/Mn_3_O_4_ NPs tended to be smaller than MnO NPs. To further explore the impact of NP size and chemical composition on Mn^2+^ release rate and MRI signal, the ideal MnO NPs synthesized in this study (32 nm) were compared with smaller MnO NPs (19 nm) and smaller Mn_3_O_4_ NPs (17 nm), with and without PLGA encapsulation. As predicted, the smallest unencapsulated MnO NPs released the most Mn^2+^ ions at pH 5 and 6.5 and led to the greatest reduction in T_1_ longitudinal relaxation time, with the highest MRI signal. PLGA encapsulation of large and small MnO NPs reduced the trends observed with unencapsulated MnO NPs possibly due to variability of metal oxide loading within individual NPs. With and without PLGA coating, Mn_3_O_4_ NPs were consistently the least effective MRI contrast agents; therefore, it is recommended to utilize MnO over Mn_3_O_4_ for MRI applications to expedite Mn^2+^ release and the resulting MRI signal produced. Future studies will explore varying the chemical reactant ratios to further decrease NP size and polydispersity, and using novel surface functionalization to enhance MnO NP endocytosis into cancer cells to maximize MRI contrast through Mn^2+^ generation.

## Supporting information

S1 Fig Temperature profiles of MnO NP synthesis. Reactant mixtures were heated from room temperature to 60°C over 30 minutes and then to 300°C using two different temperature ramps of a) 20°C/min or b) 10°C/min. Both temperature profiles show an aging temperature at 300°C for 30 minutes prior to cooling. Note how the temperatures measured during the experiments (red circles) closely match the theoretical programmed settings for the temperature controller (black lines), indicating precise control of MnO NP fabrication conditions.
(TIF)

S2 Fig Size distributions of the diameter of MnO NPs produced using the following temperature profiles: a) 5 min at 300°C with a 20°C/min vs. 10°C/min ramp, b) 15 min at 300°C with a 20°C/min vs. 10°C/min ramp, and c) 30 min at 300°C with a 20°C/min vs. 10°C/min ramp. MnO NP diameter increases as the ramping rate decreases and aging time at 300°C increases. The average size for each distribution is shown in S1 Table.
(TIF)

S3 Fig EDS spectra of the MnO NP samples with the following temperature profiles: a) 5 min at 300°C with 20°C/min ramp, b) 5 min at 300°C with 10°C/min ramp, c) 15 min at 300°C with 20°C/min ramp, d) 15 min at 300°C with 10°C/min ramp, e) 30 min at 300 ^o^C with 20°C/min ramp, and f) 30 min at 300°C with 10°C/min ramp. EDS confirmed the presence of Mn and O elements in NP samples.
(TIF)

S4 Fig FTIR spectrum of oleylamine. Asterisks represent oleyl groups, while squares represent amine groups.
(TIF)

S5 Fig Cumulative release of Mn^2+^ from MnO NPs over 24 hr after incubation in PBS pH 7.4 (dotted line), 20 mM citrate buffer pH 6.5 (dashed line), and 20 mM citrate buffer pH 5 (solid line). Controlled release curves are shown for MnO NPs generated with the following temperature profiles: a) 5 min at 300°C with 20°C/min ramp, b) 5 min at 300°C with 10°C/min ramp, c) 15 min at 300°C with 20°C/min ramp, d) 15 min at 300°C with 10°C/min ramp, and e) 30 min at 300°C with 10°C/min ramp. Mn^2+^ release increased with a decrease in pH. Time points are shown for 1, 2, 4, 8 and 24 hr. Error bars show mean ± standard deviation.
(TIF)

S6 Fig XRD and TEM of small Mn_3_O_4_ NPs and small MnO NPs. XRD spectra of a) 17 nm Mn_3_O_4_ NPs and b) 19 nm MnO NPs. The standard diffraction peaks for known c) Mn_3_O_4_ and d) MnO are shown from X’Pert HighScore. Through comparing with the standard diffraction peaks, Mn_3_O_4_ NPs are 73–100% Mn_3_O_4_ composition and MnO NPs are 67–73% MnO composition. TEM images of e) 17 nm Mn_3_O_4_ and f) 19 nm MnO NPs. NPs are smaller in size compared to Fig 2 and have a lower size variation. Scale bar is 50 nm.
(TIF)

S7 Fig MRI properties of Mn^2+^ standard curve solutions and Mn^2+^ supernatants collected from dissolving MnO and Mn_3_O_4_ NPs. a) r_1_ values for free Mn^2+^ in 20 mM citrate buffer pH 5 (black), 20 mM citrate buffer pH 6.5 (blue), and PBS pH 7.4 (red). T_1_-weighted MRI images shown in b-e) were acquired at 1 T with a 400 ms repetition time. T_1_ MRI of increasing Mn^2+^ concentrations in b) 20 mM citrate buffer pH 5, c) 20 mM citrate buffer pH 6.5, d) PBS pH 7.4. e) shows T_1_ MRI images of supernatants collected from small Mn_3_O_4_ (17 nm), small MnO (19 nm) and large MnO (32 nm) NPs suspended in pH 5 citrate buffer for 24 hours. MRI signal enhancement is greatest from small MnO NPs and least from small Mn_3_O_4_ NPs.
(TIF)

S8 Fig TEM images of PLGA encapsulated NPs formed by single emulsion. Three different types of metal oxide NPs were coated with PLGA including a) 17 nm Mn_3_O_4_, b) 19 nm MnO, and c) 32 nm MnO. Metal oxide NPs can be visualized as dark circles inside of the PLGA. NP loading capacity was ~30%. Scale bars are 100 nm.
(TIF)

S9 Fig FTIR spectra of PLGA encapsulated NPs: a) PLGA Mn_3_O_4_ (17 nm), b) PLGA MnO (19 nm), and c) PLGA MnO (32 nm). All NPs possess the characteristic peaks of PLGA, represented by circles, as shown in S10 Fig.
(TIF)

S10 Fig FTIR spectrum of PLGA. Circles represent characteristic peaks of PLGA. The peaks at 2993 cm^−1^ and 2989 cm^−1^ show the C–H stretch of CH_2_, and C–H stretch of–C–H–, respectively. The peak at 1751 cm^−1^ is assigned to the C = O stretching vibration of the ester bond and 1165–1087 cm^−1^ corresponds to the C–O stretching.
(DOCX)

S11 Fig Size distributions of PLGA NP diameters by DLS analysis: a) PLGA Mn3O4 (17 nm), b) PLGA MnO (19 nm), and c) PLGA MnO (32 nm). Highest peak for NP diameters is in the 220 to 255 nm bin size range.
(TIF)

S12 Fig MRI properties of Mn^2+^ supernatants collected from dissolving PLGA MnO and PLGA Mn_3_O_4_ NPs. T_1_ MRI images of supernatants collected from PLGA Mn_3_O_4_ (17 nm), PLGA MnO (19 nm) and PLGA MnO (32 nm) NPs suspended in pH 5 citrate buffer, pH 6.5 citrate buffer, and pH 7.4 PBS for 24 hours. MRI signal enhancement is significantly greater from PLGA MnO NPs compared to PLGA Mn_3_O_4_ NPs.
(TIF)

S1 Table Total reaction time, average diameter and PDI of MnO NPs for each temperature condition. (TIF)

S2 Table Total amount of Mn^2+^ released (ave ± stdev) from MnO NPs over 24 hr at different pH. (TIF)

S3 Table Concentration of Mn^2+^ obtained from controlled release of NPs at 24 hr analyzed by ICP-OES and MRI. (TIF)

S4 Table Loading capacity of PLGA NPs (mg Mn_x_O_y_/mg NP) by ICP-OES. (TIF)
