# Supplementary figures and images for: Tuning the size and composition of manganese oxide nanoparticles through varying temperature ramp and aging time
Source: PLoS One. 2020 Sep 18;15(9):e0239034. doi: 10.1371/journal.pone.0239034 (PMC7500698; doi:10.1371/journal.pone.0239034)

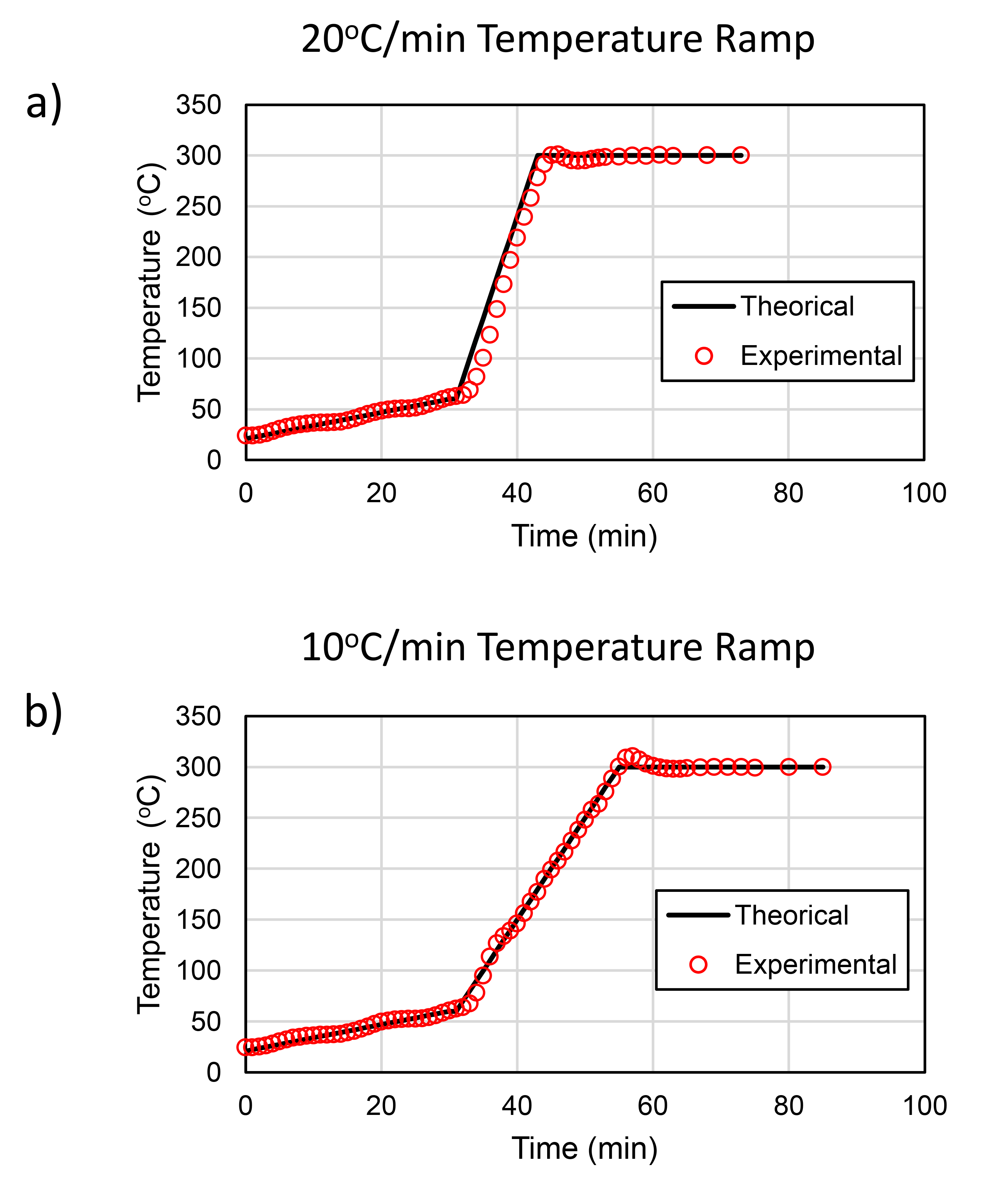

Supplement: S1 Fig — Reactant mixtures were heated from room temperature to 60°C over 30 minutes and then to 300°C using two different temperature ramps of a) 20°C/min or b) 10°C/min. Both temperature profiles show an aging temperature at 300°C for 30 minutes prior to cooling. Note how the temperatures measured during the experiments (red circles) closely match the theoretical programmed settings for the temperature controller (black lines), indicating precise control of MnO NP fabrication conditions. (TIF) [file pone.0239034.s001.tif]

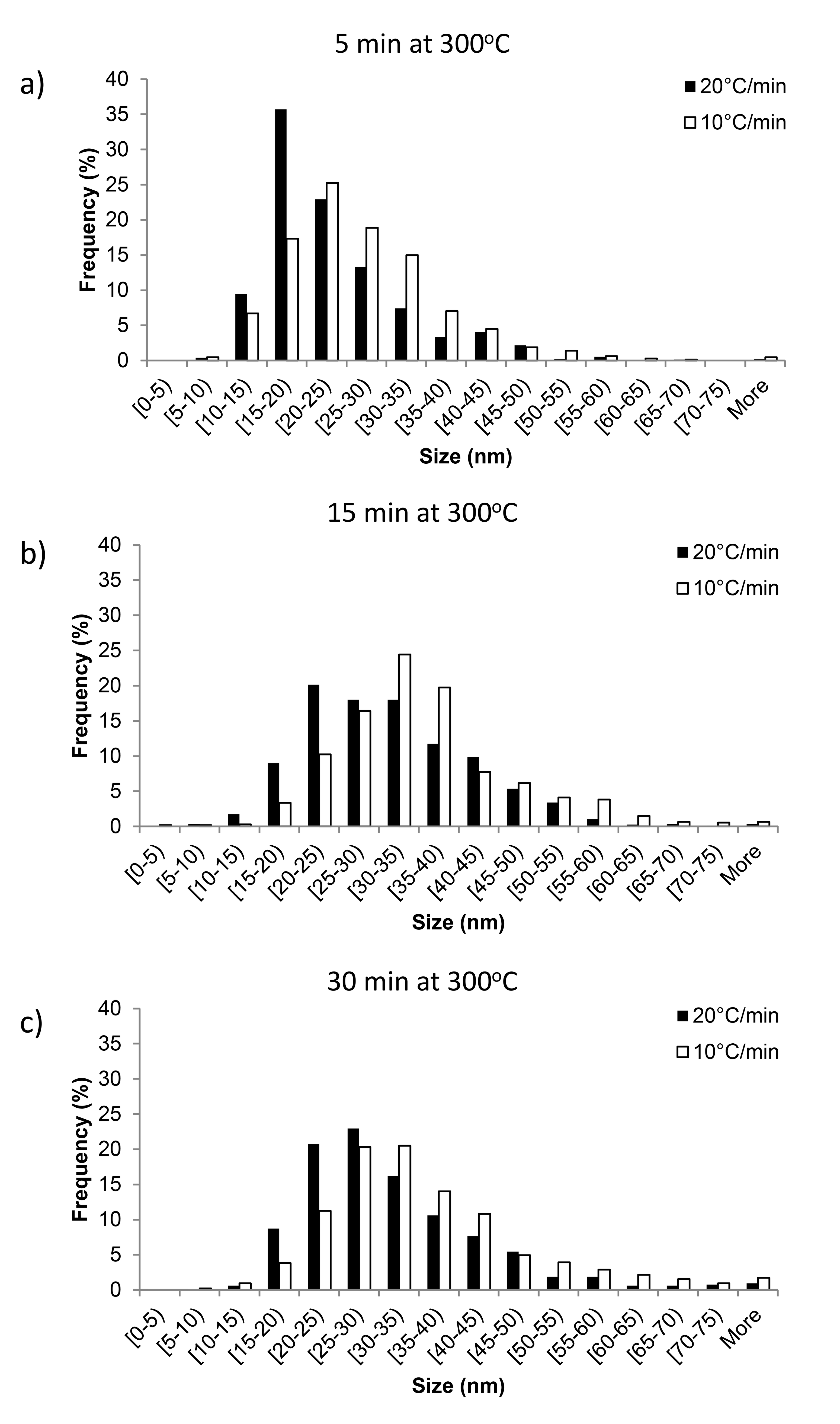

Supplement: S2 Fig — Size distributions of the diameter of MnO NPs produced using the following temperature profiles: a) 5 min at 300°C with a 20°C/min vs. 10°C/min ramp, b) 15 min at 300°C with a 20°C/min vs. 10°C/min ramp, and c) 30 min at 300°C with a 20°C/min vs. 10°C/min ramp. MnO NP diameter increases as the ramping rate decreases and aging time at 300°C increases. The average size for each distribution is shown in S1 Table. (TIF) [file pone.0239034.s002.tif]

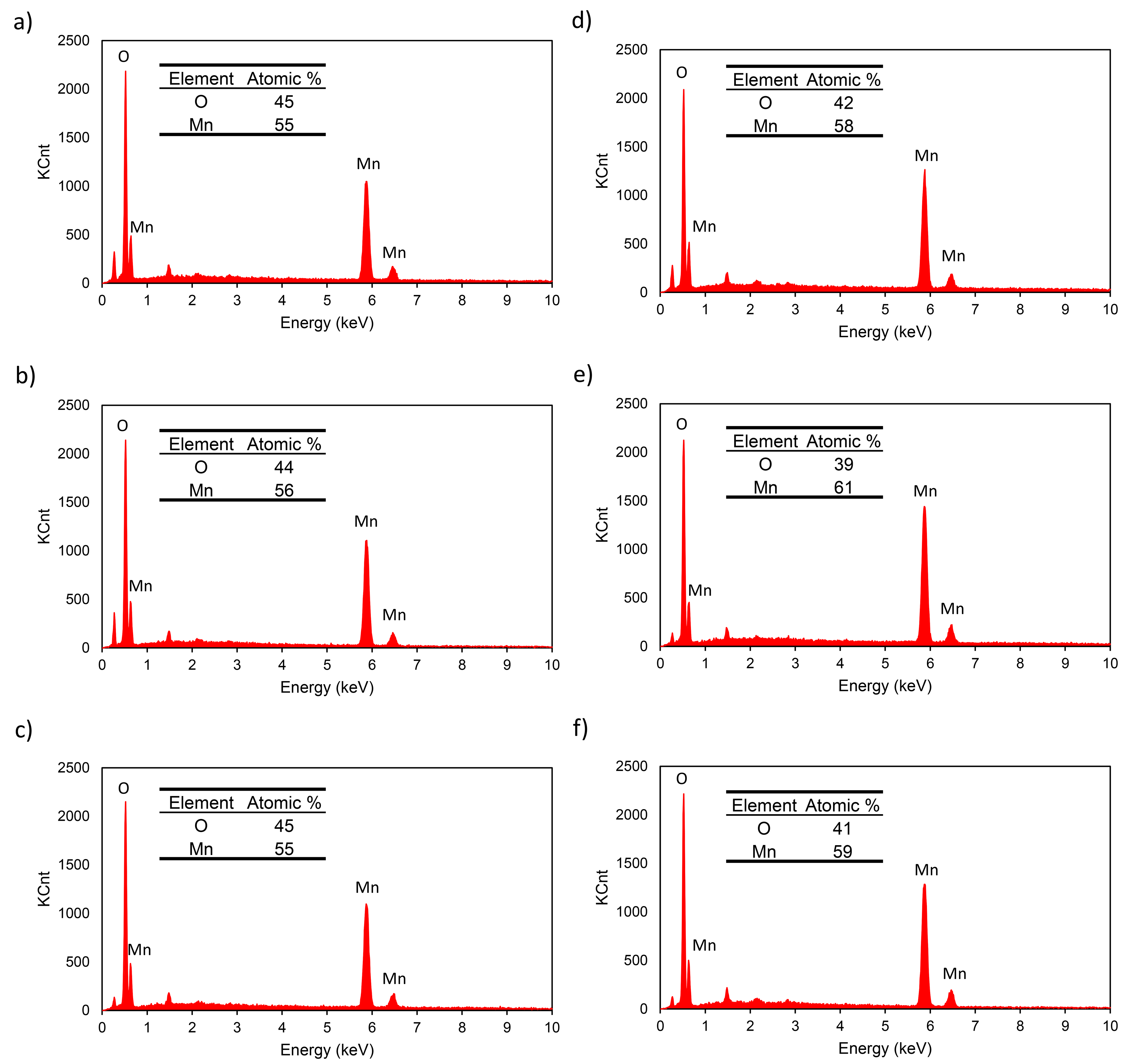

Supplement: S3 Fig — EDS spectra of the MnO NP samples with the following temperature profiles: a) 5 min at 300°C with 20°C/min ramp, b) 5 min at 300°C with 10°C/min ramp, c) 15 min at 300°C with 20°C/min ramp, d) 15 min at 300°C with 10°C/min ramp, e) 30 min at 300 oC with 20°C/min ramp, and f) 30 min at 300°C with 10°C/min ramp. EDS confirmed the presence of Mn and O elements in NP samples. (TIF) [file pone.0239034.s003.tif]

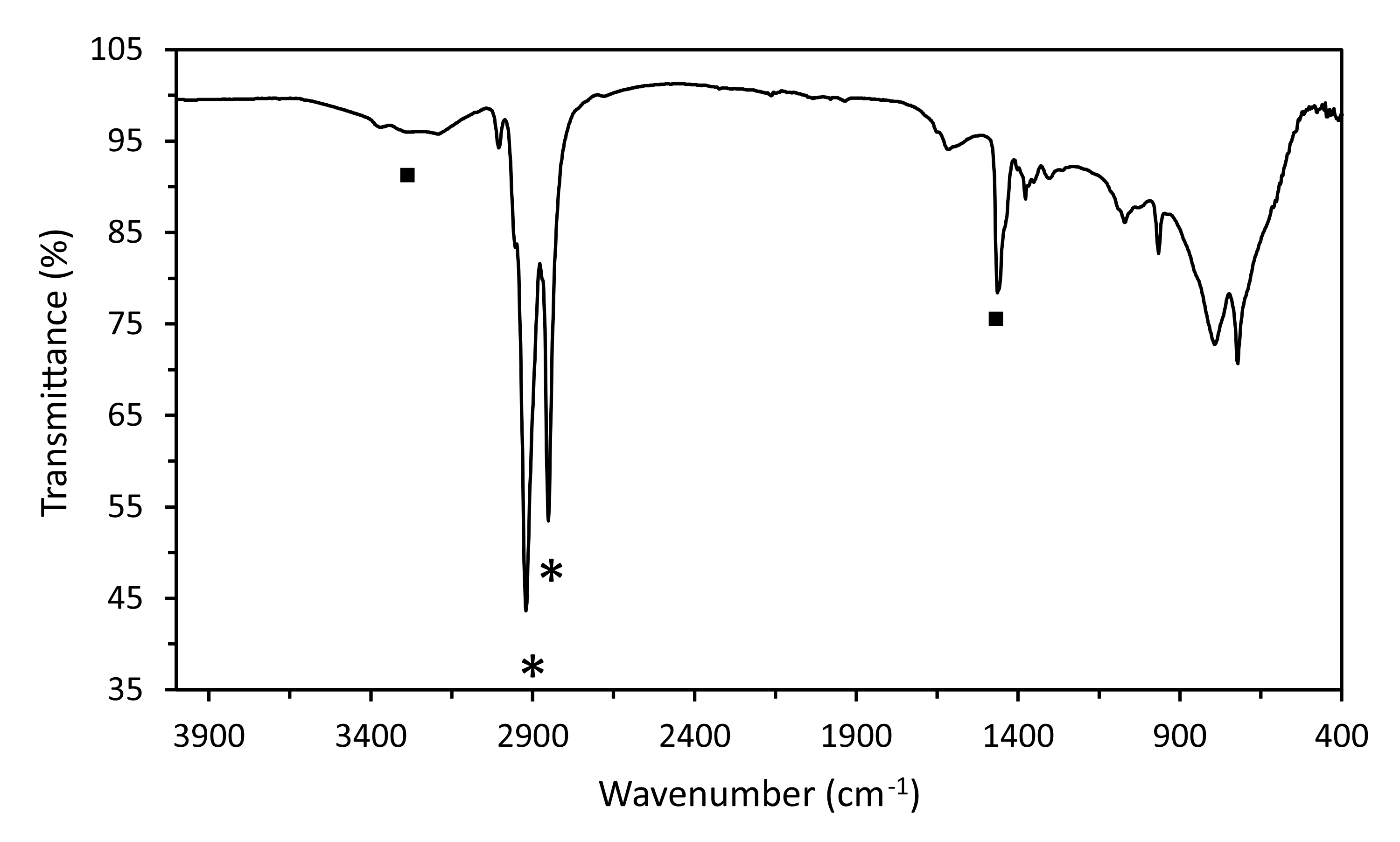

Supplement: S4 Fig — Asterisks represent oleyl groups, while squares represent amine groups. (TIF) [file pone.0239034.s004.tif]

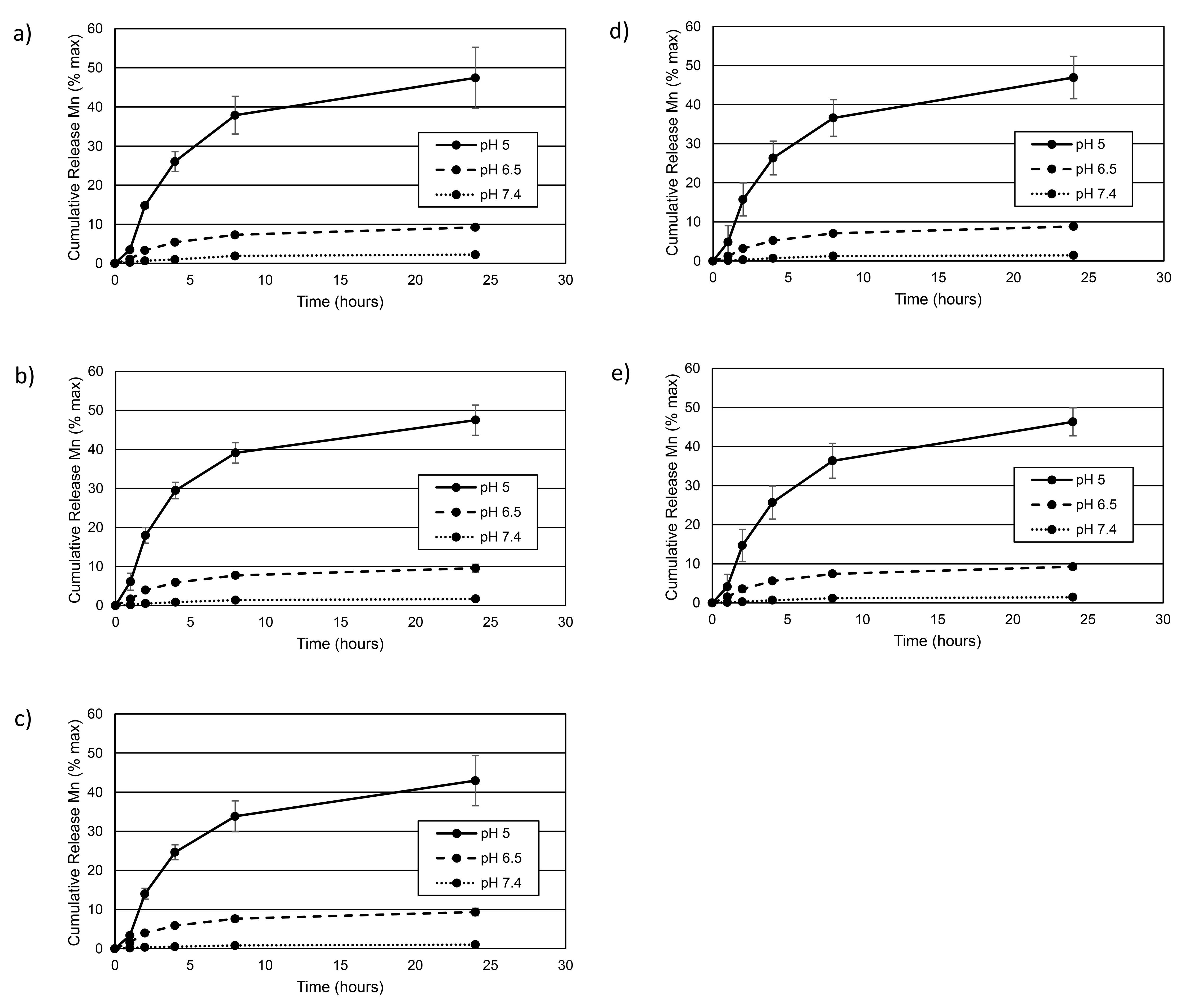

Supplement: S5 Fig — Controlled release curves are shown for MnO NPs generated with the following temperature profiles: a) 5 min at 300°C with 20°C/min ramp, b) 5 min at 300°C with 10°C/min ramp, c) 15 min at 300°C with 20°C/min ramp, d) 15 min at 300°C with 10°C/min ramp, and e) 30 min at 300°C with 10°C/min ramp. Mn2+ release increased with a decrease in pH. Time points are shown for 1, 2, 4, 8 and 24 hr. Error bars show mean ± standard deviation. (TIF) [file pone.0239034.s005.tif]

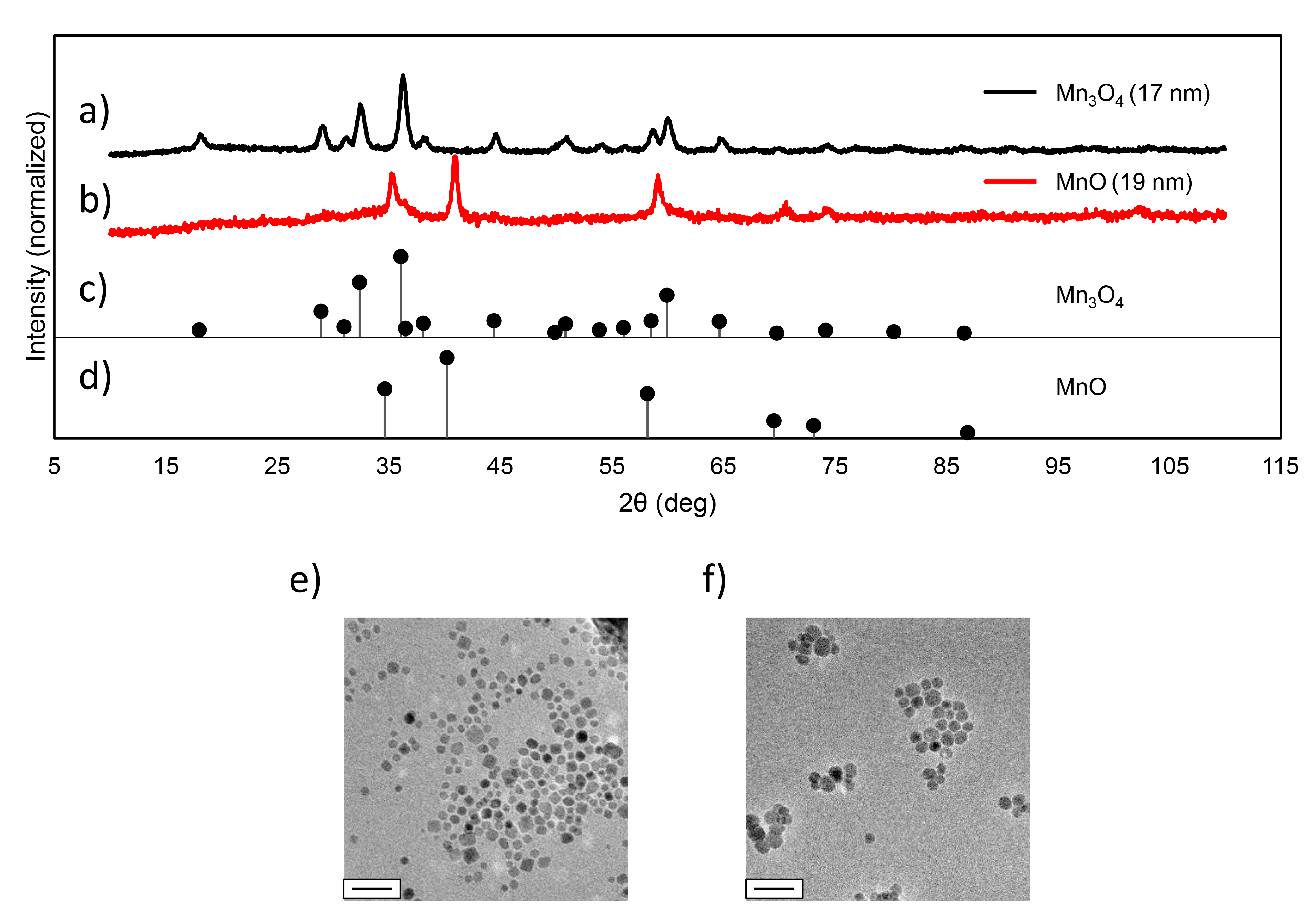

Supplement: S6 Fig — XRD spectra of a) 17 nm Mn3O4 NPs and b) 19 nm MnO NPs. The standard diffraction peaks for known c) Mn3O4 and d) MnO are shown from X’Pert HighScore. Through comparing with the standard diffraction peaks, Mn3O4 NPs are 73–100% Mn3O4 composition and MnO NPs are 67–73% MnO composition. TEM images of e) 17 nm Mn3O4 and f) 19 nm MnO NPs. NPs are smaller in size compared to Fig 2 and have a lower size variation. Scale bar is 50 nm. (TIF) [file pone.0239034.s006.tif]

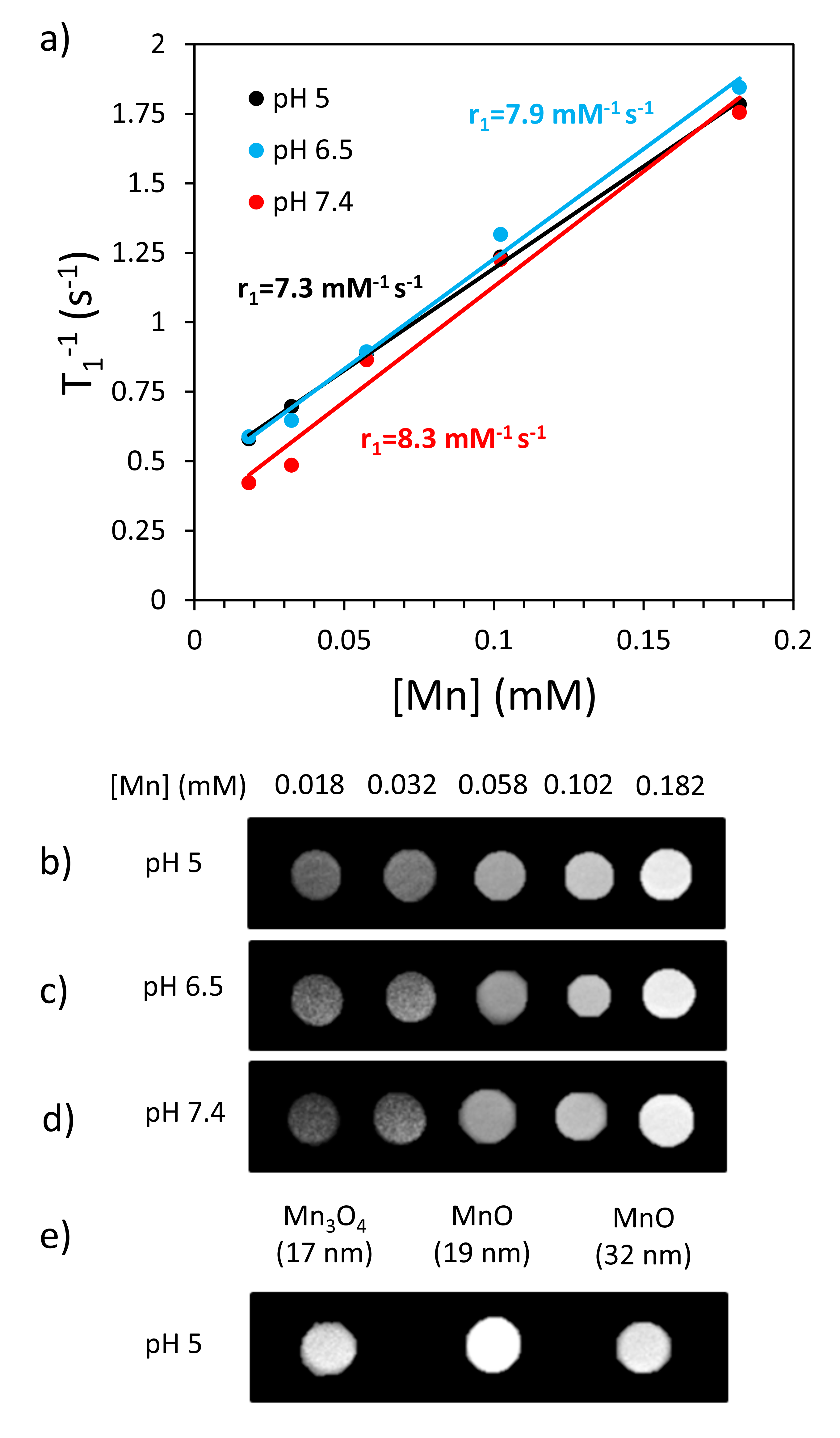

Supplement: S7 Fig — a) r1 values for free Mn2+ in 20 mM citrate buffer pH 5 (black), 20 mM citrate buffer pH 6.5 (blue), and PBS pH 7.4 (red). T1-weighted MRI images shown in b-e) were acquired at 1 T with a 400 ms repetition time. T1 MRI of increasing Mn2+ concentrations in b) 20 mM citrate buffer pH 5, c) 20 mM citrate buffer pH 6.5, d) PBS pH 7.4. e) shows T1 MRI images of supernatants collected from small Mn3O4 (17 nm), small MnO (19 nm) and large MnO (32 nm) NPs suspended in pH 5 citrate buffer for 24 hours. MRI signal enhancement is greatest from small MnO NPs and least from small Mn3O4 NPs. (TIF) [file pone.0239034.s007.tif]

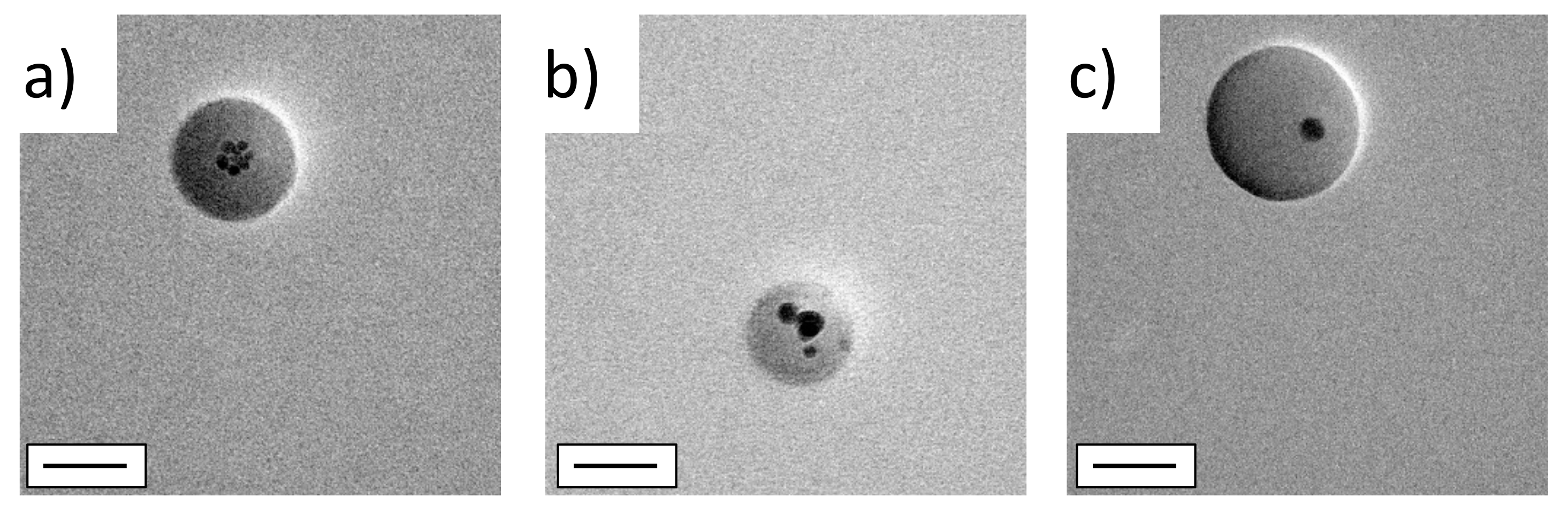

Supplement: S8 Fig — Three different types of metal oxide NPs were coated with PLGA including a) 17 nm Mn3O4, b) 19 nm MnO, and c) 32 nm MnO. Metal oxide NPs can be visualized as dark circles inside of the PLGA. NP loading capacity was ~30%. Scale bars are 100 nm. (TIF) [file pone.0239034.s008.tif]

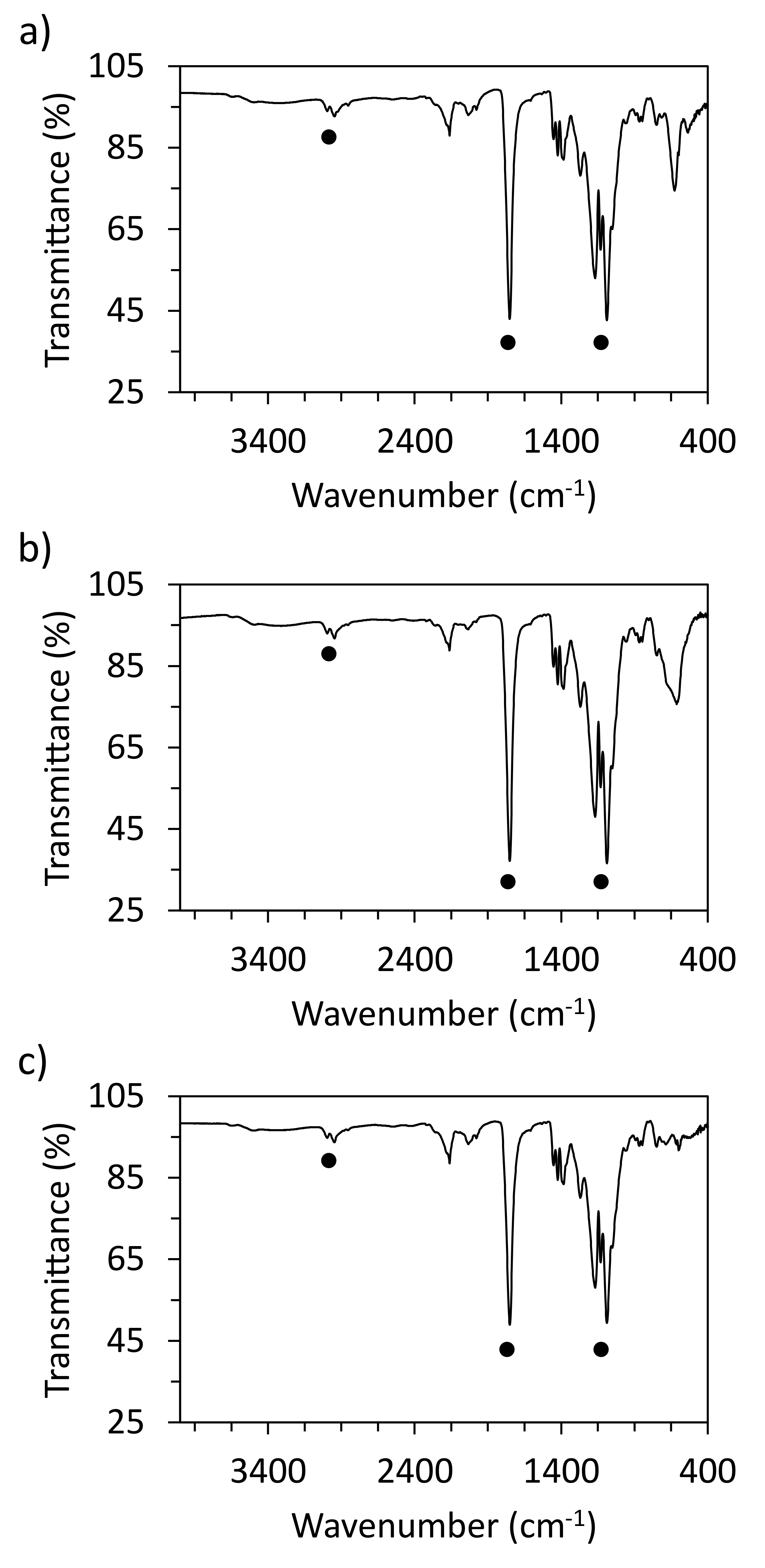

Supplement: S9 Fig — FTIR spectra of PLGA encapsulated NPs: a) PLGA Mn3O4 (17 nm), b) PLGA MnO (19 nm), and c) PLGA MnO (32 nm). All NPs possess the characteristic peaks of PLGA, represented by circles, as shown in S10 Fig. (TIF) [file pone.0239034.s009.tif]

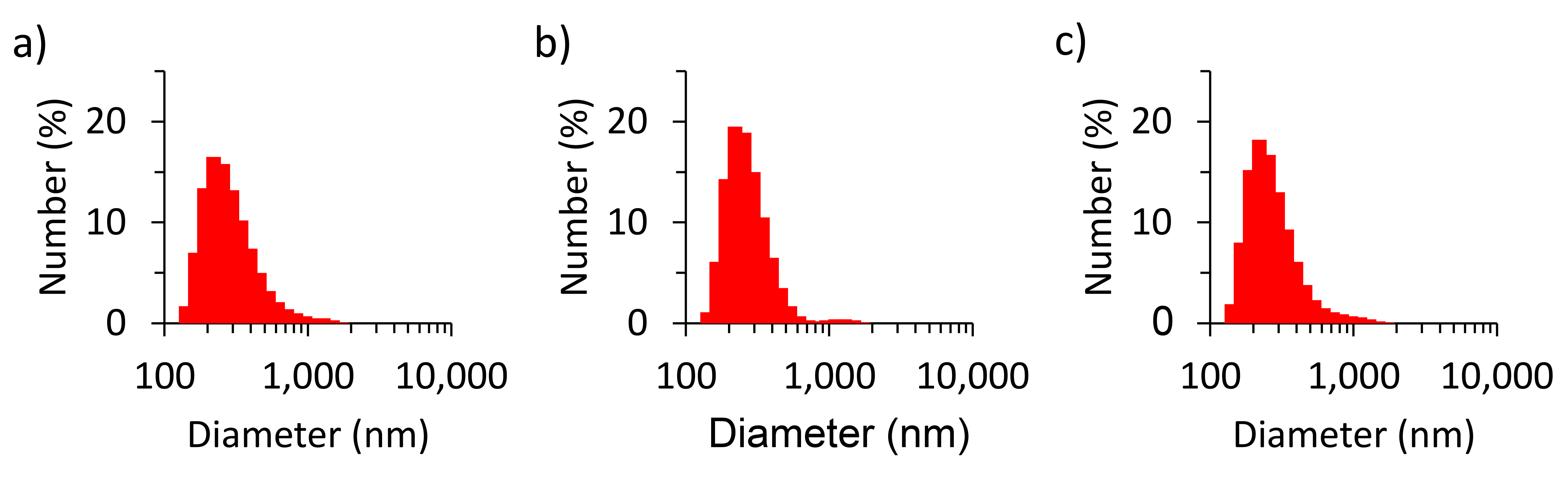

Supplement: S11 Fig — Size distributions of PLGA NP diameters by DLS analysis: a) PLGA Mn3O4 (17 nm), b) PLGA MnO (19 nm), and c) PLGA MnO (32 nm). Highest peak for NP diameters is in the 220 to 255 nm bin size range. (TIF) [file pone.0239034.s011.tif]

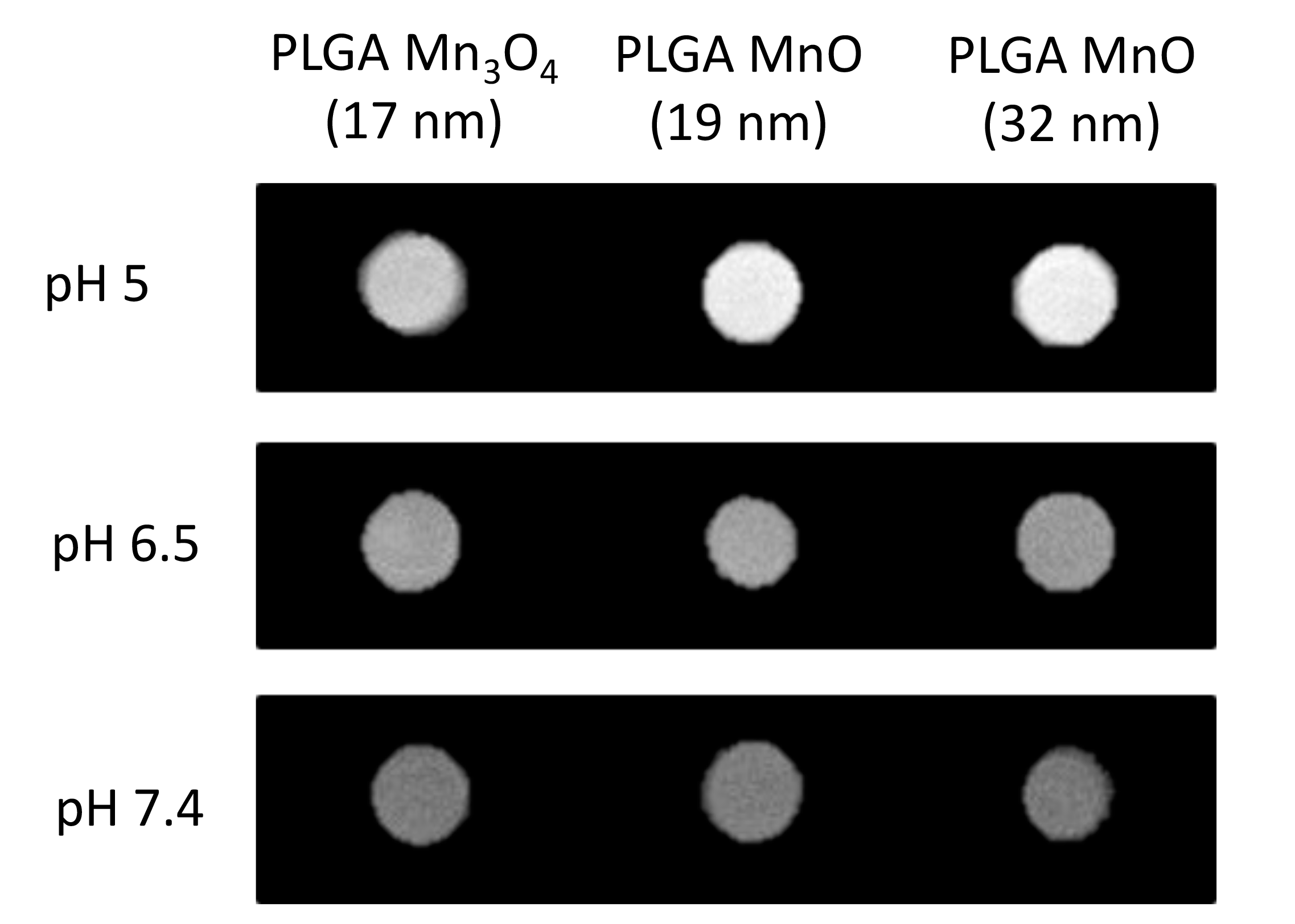

Supplement: S12 Fig — T1 MRI images of supernatants collected from PLGA Mn3O4 (17 nm), PLGA MnO (19 nm) and PLGA MnO (32 nm) NPs suspended in pH 5 citrate buffer, pH 6.5 citrate buffer, and pH 7.4 PBS for 24 hours. MRI signal enhancement is significantly greater from PLGA MnO NPs compared to PLGA Mn3O4 NPs. (TIF) [file pone.0239034.s012.tif]

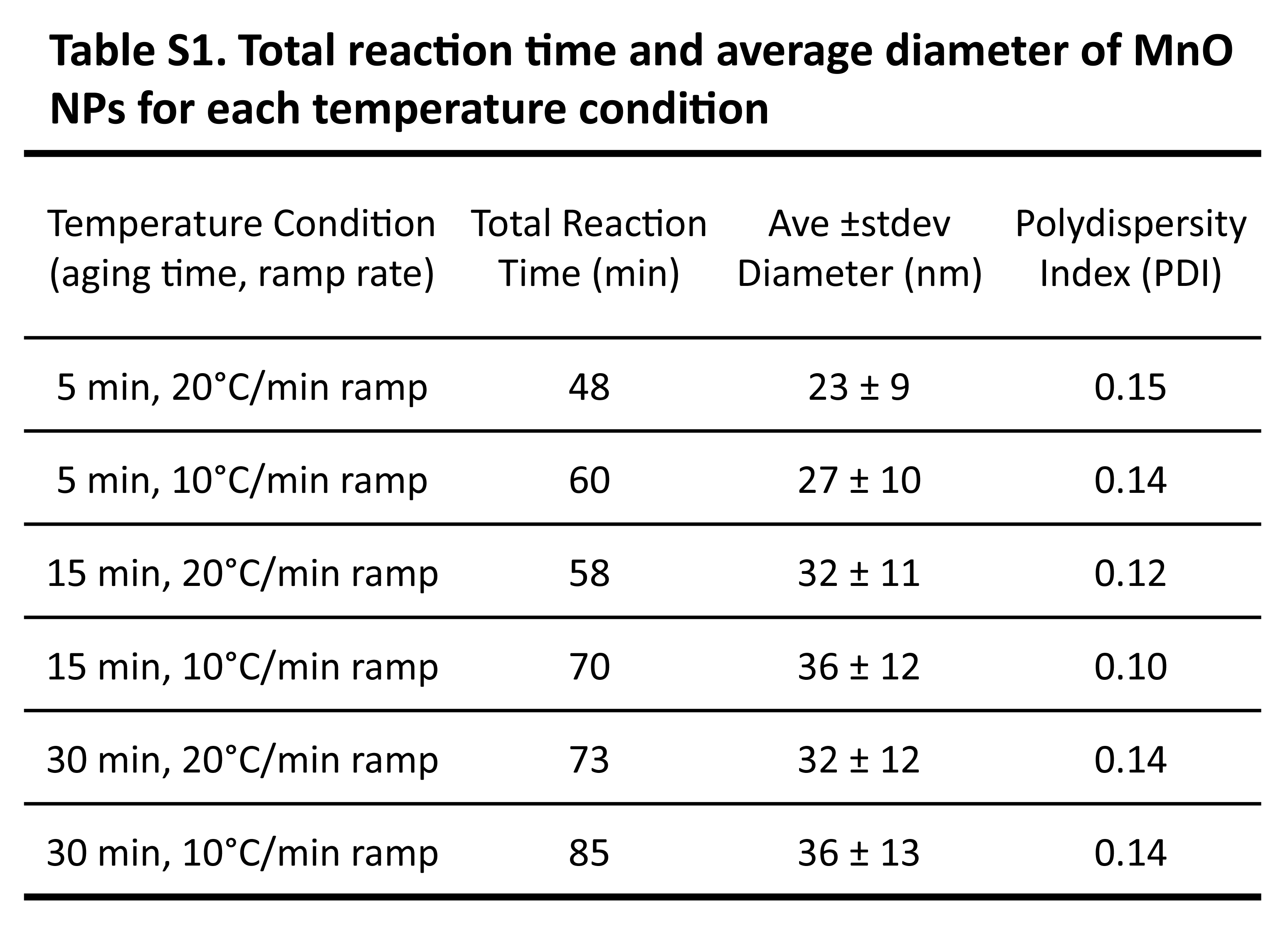

Supplement: S1 Table — (TIF) [file pone.0239034.s013.tif]

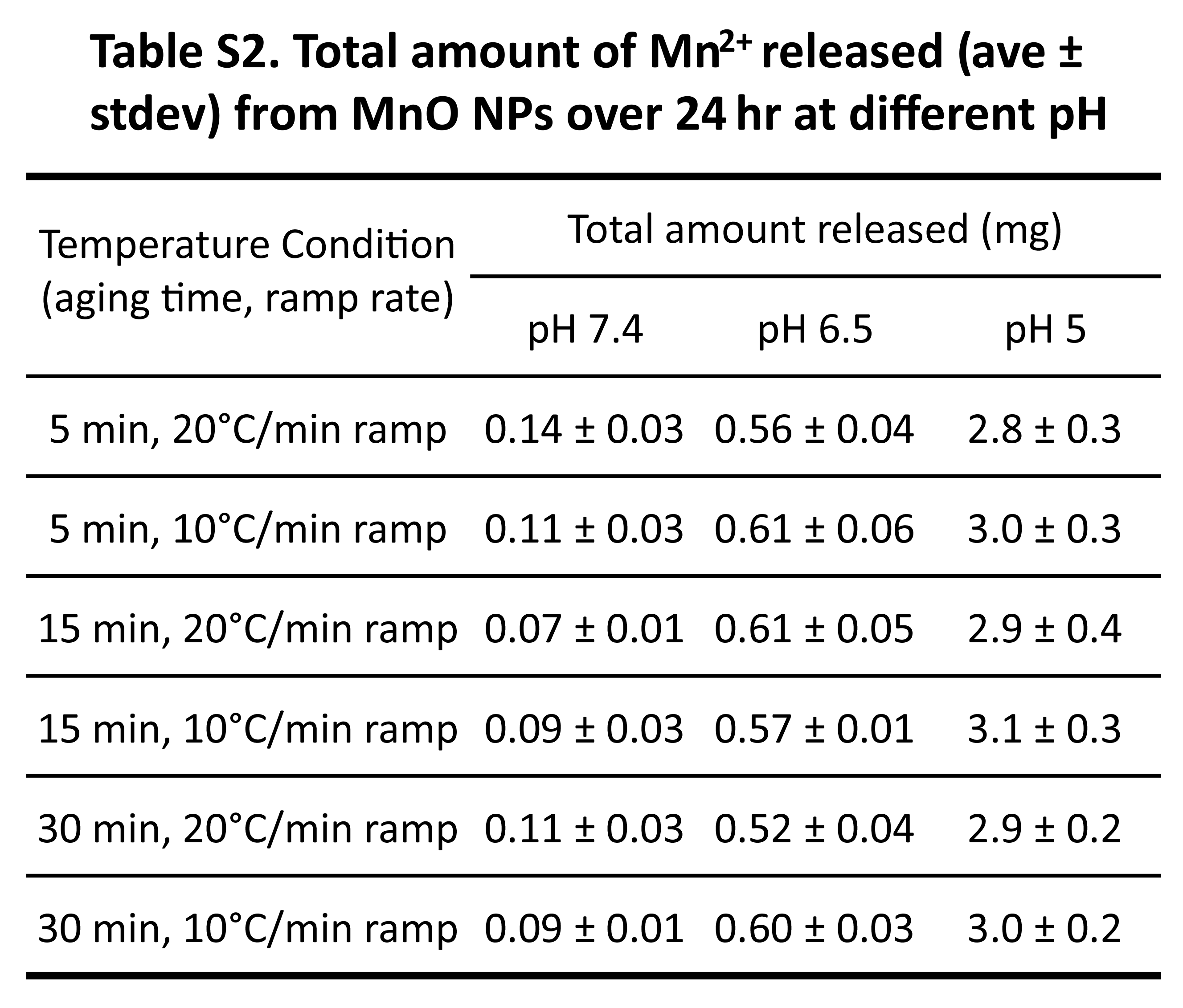

Supplement: S2 Table — (TIF) [file pone.0239034.s014.tif]

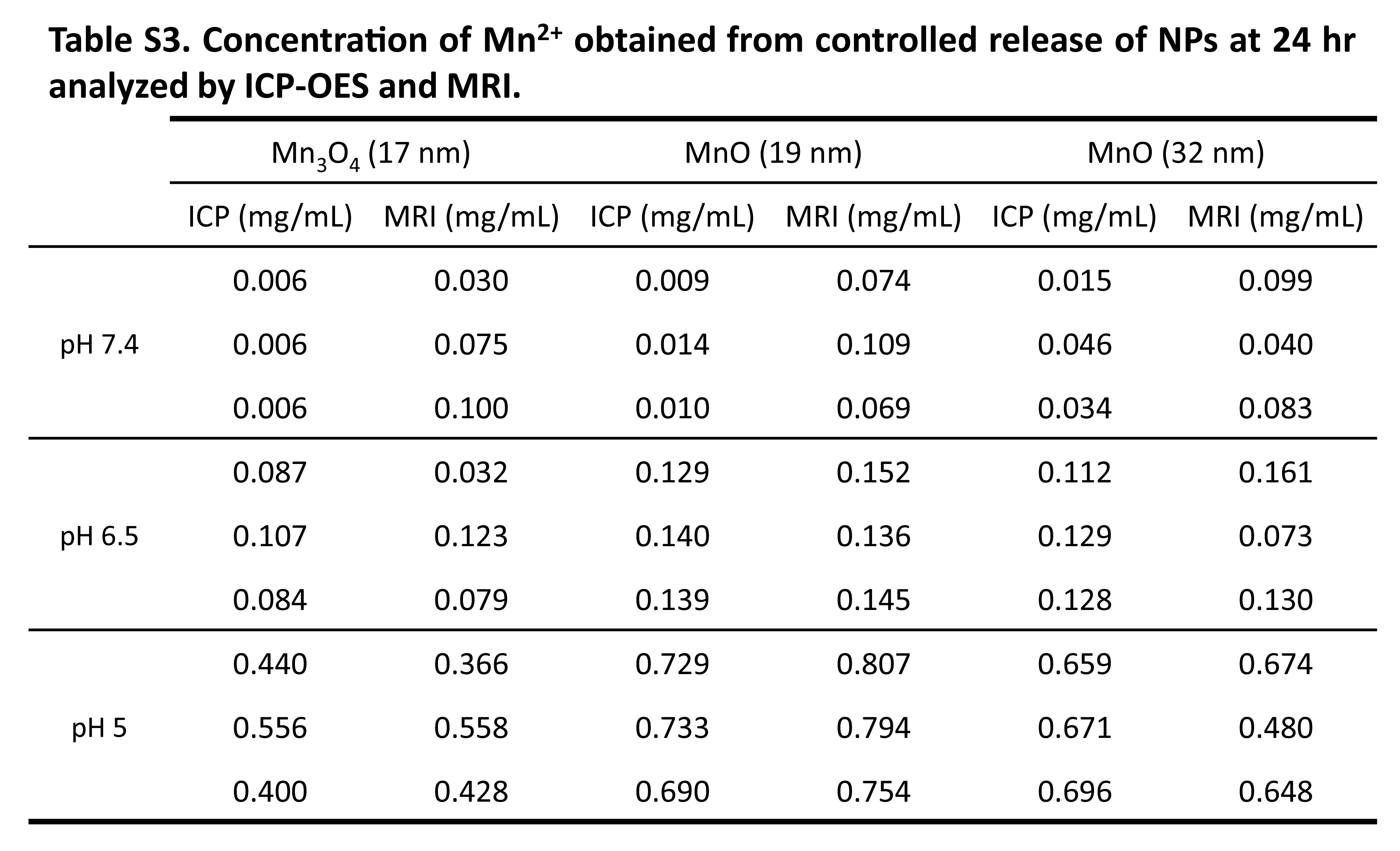

Supplement: S3 Table — (TIF) [file pone.0239034.s015.tif]

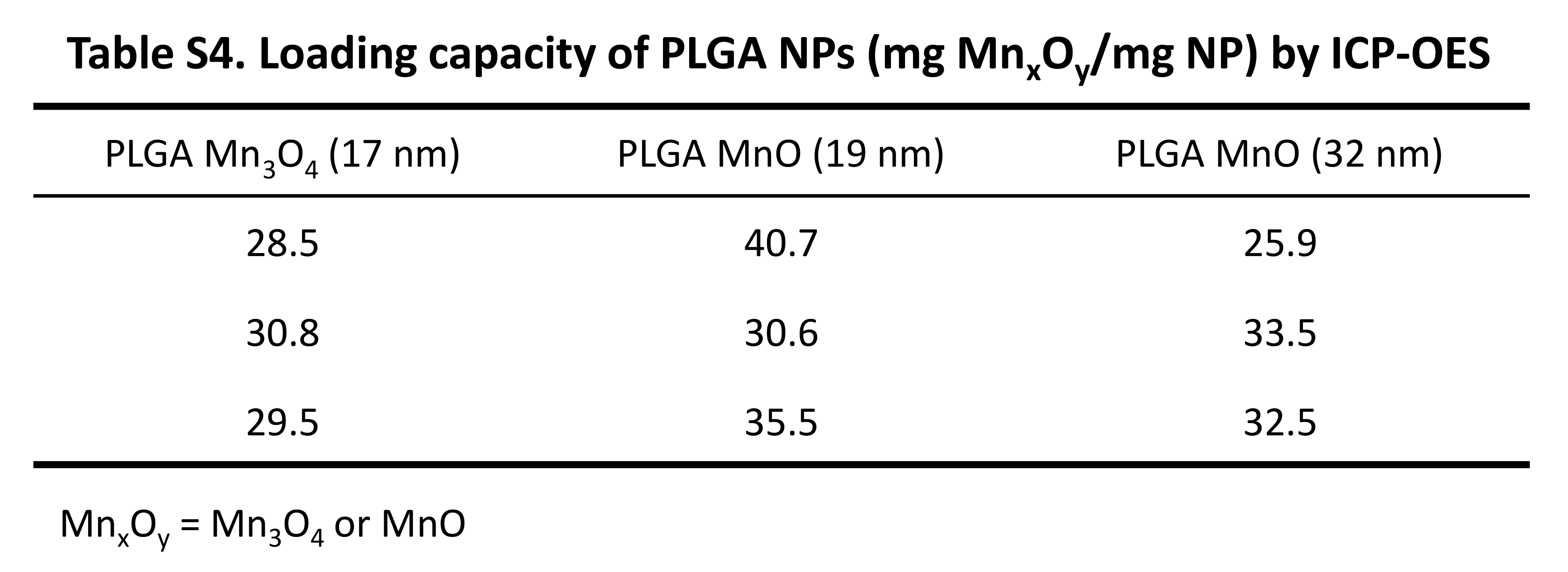

Supplement: S4 Table — (TIF) [file pone.0239034.s016.tif]
