# Supplementary material for: Tuning the size and composition of manganese oxide nanoparticles through varying temperature ramp and aging time
Source: PLoS One. 2020 Sep 18;15(9):e0239034. doi: 10.1371/journal.pone.0239034 (PMC7500698; doi:10.1371/journal.pone.0239034)
Supplement: S10 Fig — Circles represent characteristic peaks of PLGA. The peaks at 2993 cm−1 and 2989 cm−1 show the C–H stretch of CH2, and C–H stretch of–C–H–, respectively. The peak at 1751 cm−1 is assigned to the C = O stretching vibration of the ester bond and 1165–1087 cm−1 corresponds to the C–O stretching. (DOCX) [file pone.0239034.s010.docx]

**Fig S10. FTIR spectrum of PLGA.** Circles represent characteristic peaks of PLGA. The peaks at 2993 cm^−1^ and 2989 cm^−1^ show the C–H stretch of CH_2_, and C–H stretch of –C–H–, respectively. The peak at 1751 cm^−1^ is assigned to the C=O stretching vibration of the ester bond and 1165 - 1087 cm^−1^ corresponds to the C–O stretching[1-3].


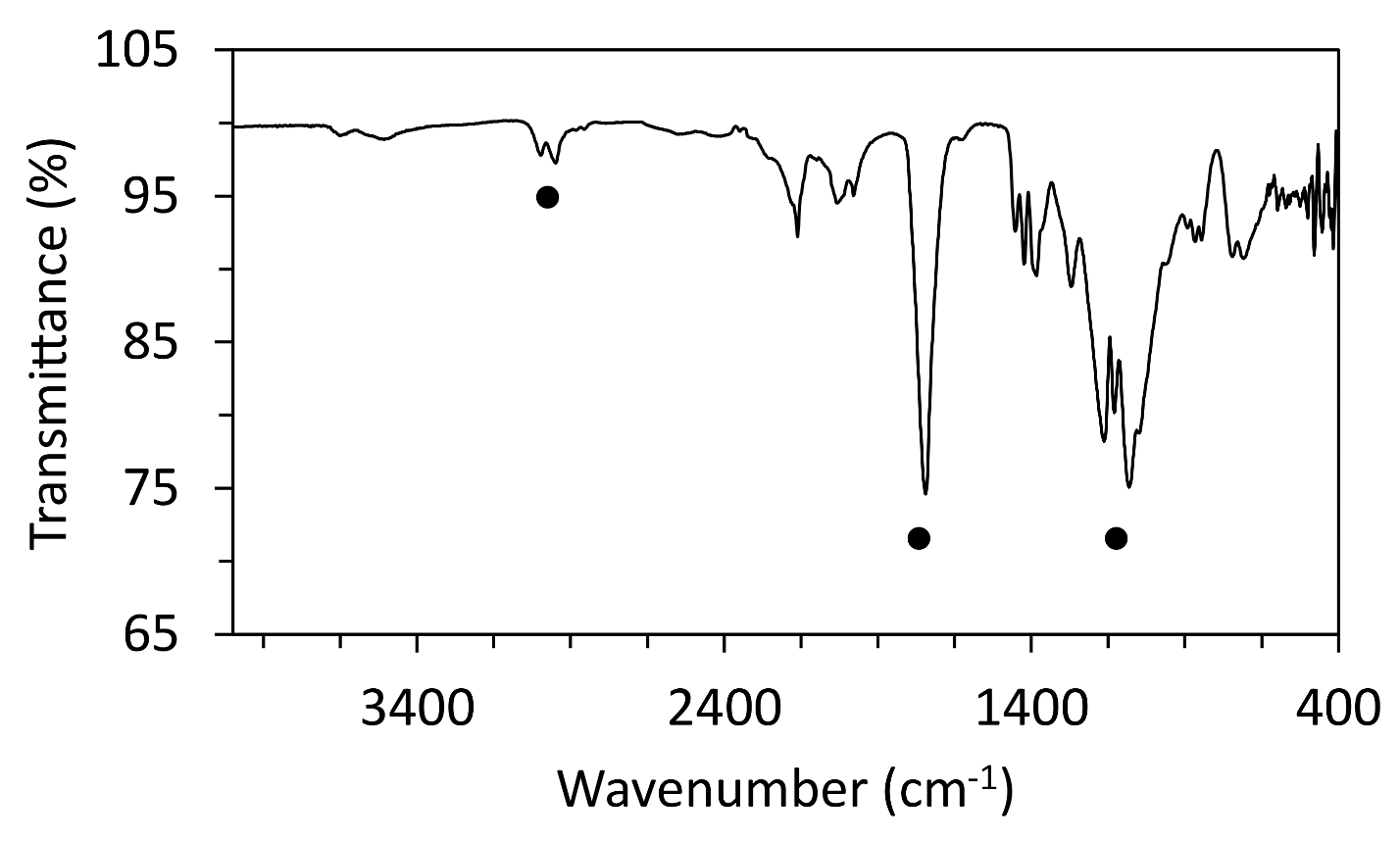


**References**

1. Wang H, Zhao Y, Wu Y, Hu Y, Nan K, Nie G, et al. Enhanced anti-tumor efficacy by co-delivery of doxorubicin and paclitaxel with amphiphilic methoxy PEG-PLGA copolymer nanoparticles. Biomaterials. 2011 Nov 1;32(32):8281–90.

2. Arasoglu T, Derman S, Mansuroglu B. Comparative evaluation of antibacterial activity of caffeic acid phenethyl ester and PLGA nanoparticle formulation by different methods. Nanotechnology. 2016 Jan 15;27(2):025103.

3. Jiang P, Yu D, Zhang W, Mao Z, Gao C. Influence of bovine serum albumin coated poly(lactic-co-glycolic acid) particles on differentiation of mesenchymal stem cells. RSC Adv. 2015 May 5;5(51):40924–31.
